# A new *in vivo* model of intestinal colonization using *Zophobas morio* larvae: testing hyperepidemic ESBL- and carbapenemase-producing *Escherichia coli* clones

**DOI:** 10.3389/fmicb.2024.1381051

**Published:** 2024-04-10

**Authors:** Yasmine Eddoubaji, Claudia Aldeia, Edgar I. Campos-Madueno, Aline I. Moser, Cindy Kundlacz, Vincent Perreten, Markus Hilty, Andrea Endimiani

**Affiliations:** ^1^Institute for Infectious Diseases (IFIK), University of Bern, Bern, Switzerland; ^2^Graduate School of Cellular and Biomedical Sciences, University of Bern, Bern, Switzerland; ^3^Institute of Veterinary Bacteriology, University of Bern, Bern, Switzerland

**Keywords:** ESBL, carbapenemase, bacteriophages, *in vivo*, colonization, ST131, ST410, ST167

## Abstract

Finding strategies for decolonizing gut carriers of multidrug-resistant *Escherichia coli* (MDR-*Ec*) is a public-health priority. In this context, novel approaches should be validated in preclinical *in vivo* gut colonization models before being translated to humans. However, the use of mice presents limitations. Here, we used for the first time *Zophobas morio* larvae to design a new model of intestinal colonization (28-days duration, T28). Three hyperepidemic MDR-*Ec* producing extended-spectrum β-lactamases (ESBLs) or carbapenemases were administered via contaminated food to larvae for the first 7 days (T7): *Ec*-4901.28 (ST131, CTX-M-15), *Ec*-042 (ST410, OXA-181) and *Ec*-050 (ST167, NDM-5). Growth curve analyses showed that larvae became rapidly colonized with all strains (T7, ~10^6–7^ CFU/mL), but bacterial load remained high after the removal of contaminated food only in *Ec*-4901.28 and *Ec*-042 (T28, ~10^3–4^ CFU/mL). Moreover, larvae receiving a force-feeding treatment with *INTESTI bacteriophage* cocktail (on T7 and T10 via gauge needle) were decolonized by *Ec*-4901.28 (*INTESTI*-susceptible); however, *Ec*-042 and *Ec*-050 (*INTESTI*-resistant) did not. Initial microbiota (before administering contaminated food) was very rich of bacterial genera (e.g., *Lactococcus*, *Enterococcus, Spiroplasma*), but patterns were heterogeneous (Shannon diversity index: range 1.1–2.7) and diverse to each other (Bray–Curtis dissimilarity index ≥30%). However, when larvae were challenged with the MDR-*Ec* with or without administering bacteriophages the microbiota showed a non-significant reduction of the diversity during the 28-day experiments. In conclusion, the *Z. morio* larvae model promises to be a feasible and high-throughput approach to study novel gut decolonization strategies for MDR-*Ec* reducing the number of subsequent confirmatory mammalian experiments.

## 1 Introduction

The spread of multidrug-resistant *Enterobacteriaceae* (MDR-*Ent*) producing plasmid-mediated extended-spectrum β-lactamases (ESBLs) of CTX-M-type and/or carbapenemases (e.g., KPC-, OXA-48-like, and NDM-types) is a global concern (Bonomo et al., 2018; Peirano and Pitout, 2019). In particular, MDR *Escherichia coli* (MDR-*Ec*) strains, which frequently belong to hyperepidemic clones [e.g., sequence types (ST)131, ST167, ST410], can easily colonize the intestinal tract of animals and people and spread successfully in both hospital and community settings (Nigg et al., 2019; Peirano and Pitout, 2019; Pitout and Finn, 2020; Moser et al., 2021; Campos-Madueno et al., 2023b; Linkevicius et al., 2023; Silva et al., 2023; Pitout et al., 2024).

This colonization phenomenon is of key importance, since gut carriers may later develop difficult-to-treat infections with high morbidity and mortality rates (Ling et al., 2021). Furthermore, intestinal carriers contribute to the spread of these MDR pathogens in different human and non-human settings *via* direct contact transmission with other subjects and/or contaminating both environment and food chain (Hilty et al., 2012; Campos-Madueno et al., 2023b; Silva et al., 2023). As a consequence, finding strategies for decolonizing gut carriers of MDR-*Ec* is a public-health priority.

Two approaches to decolonize gut carriers of MDR-*Ent* have already been implemented in the clinical context. The first is the selective digestive decontamination using broad-spectrum antibiotic(s). However, this strategy does not completely eradicate the targeted strain, leading to a disrupted colonization resistance, and may select for bacterial resistance against the antibiotic(s) used. More recently, the fecal microbiota transplantation has been implemented, but a major drawback is patient compliance. Overall, effective and standardized strategies for decolonizing gut carriers of MDR-*Ent* are still not available (Campos-Madueno et al., 2023b).

Recently, novel decolonizing strategies (e.g., use of bacteriophages, CRISPR-Cas-9-mediated curing systems) that could overcome the limitations described above have been suggested (Campos-Madueno et al., 2023b). These approaches have shown promising results in *in vitro* experiments against specific MDR-*Ent* (e.g., those producing ESBLs and/or carbapenemases), with a potentially significant clinical impact. For instance, in an *in vitro* model (chemostat bioreactor) simulating the human gut, we have demonstrated that the commercial *INTESTI bacteriophage* cocktail (Eliava BioPreparations) may decolonize human stools from a CTX-M-15-producing *E. coli* (strain *Ec*-4901.28) (Bernasconi et al., 2020). Nevertheless, our own findings, together with those of others (e.g., Laird et al., 2022), remain to be validated in preclinical *in vivo* models before they can be translated to humans.

The *in vivo* mouse model has so far represented the gold-standard to study several aspects linked to the intestinal colonization due to MDR-*Ent* (Perez et al., 2011; Javaudin et al., 2021; Stercz et al., 2021; Fang et al., 2022). However, many strong limitations can be found in terms of societal, ethical and logistical issues, which can all together lead to very long and laborious investigation periods. On the other hand, the use of an invertebrate model may provide an innovative, suitable and highly scalable substitute to the mouse in line with the Replacement, Reduction and Refinement (3Rs) strategy (Freires et al., 2017; Sneddon et al., 2017). In this context, numerous alternatives have been proposed (Freires et al., 2017), though a gut model with MDR-*Ent* has been tried only with *Danio rerio* (Zebrafish) or *Galleria mellonella* larvae (Zhang et al., 2019; Mirza et al., 2024).

*Zophobas morio* (synonym as *Z. atratus*) – a beetle belonging to the family of *Tenebrionidae* that is commonly used in the pet food industry - presents attractive characteristics that could fit very well in an MDR-*Ent* gut colonization model. In fact, its larvae possess a strong exoskeleton that could make them resistant to the frequent manipulation during experiments. Moreover, *Z. morio* larvae do not pupate under crowded conditions, thus remaining in this stage until their death (up to 6 months). Larvae can also be easily reared at room temperature supplying a large spectrum of diets (Rumbos and Athanassiou, 2021). Finally, some data indicate that they may possess a very diversified intestinal microbiota (Luo et al., 2021), although its dynamic changes in response to pathogenic bacterial challenge have not yet been investigated.

In this work, we used for the first time *Z. morio* larvae to design a new gut colonization model with MDR-*Ent*. In particular, three hyperepidemic MDR-*Ec* strains (including *Ec*-4901.28) were implemented for serial growth curve experiments coupled with microbiota dynamic analyses. The *INTESTI bacteriophage* cocktail was also tested to investigate its potential to decolonize larvae from the MDR-*Ec* strains.

## 2 Materials and methods

### 2.1 *Escherichia coli* strains used for experiments

Three previously well-characterized MDR-*Ec* strains were used for *Z. morio* larvae colonization experiments: *Ec*-4901.28 (ST131, CTX-M-15 ESBL producer) (Seiffert et al., 2013; Bernasconi et al., 2020), *Ec*-042 (ST410, OXA-181 carbapenemase producer) and *Ec*-050 (ST167, NDM-5 carbapenemase producer) (Endimiani et al., 2020). The susceptibility of these strains to the *INTESTI bacteriophage* cocktail (10 mL x 5 ampules, lot no. M2-1201) was tested using the double-layer agar method (DLA) (Clockie and Kropinski, 2009): *Ec*-4901.28 was susceptible (++; opaque lysis: turbidity throughout the cleared zone), whereas *Ec*-042 and *Ec*-050 were resistant (R; no clearing). The overall characteristics of the three MDR-*Ec* strains - including antibiotic resistance profiles, antimicrobial resistance genes (ARGs) and virulence factors – are summarized in Table 1.

**Table 1:** Phenotypic and molecular characteristics of the multidrug-resistant *E. coli* (MDR-*Ec*) strains used for colonization experiments in *Z. morio* larvae.

**Characteristics**	***Ec*-4901.28**	***Ec*-042**	***Ec*-050**
Host of isolation, sample and year of detection	Human, urine (infection), 2011	Human, stool (colonization), 2019	Human, stool (colonization), 2019
Genome assembly deposited	GCA_007714165.1^e^	GCA_008042015.2	GCA_008124425.1
Antimicrobial susceptibility tests, ASTs (MIC, mg/L)^a^			
Piperacillin-tazobactam	≤8, S	≥128, R	≥128, R
Ceftazidime	16, R	≥32, R	≥32, R
Cefotaxime	≥64, R	≥64, R	≥64, R
Cefepime	16, R	8, R	≥32, R
Aztreonam	≥32, R	≥32, R	≥32, R
Imipenem	≤1, S	≤1, S	≤1, S
Meropenem	≤1, S	≤1, S	2, S
Ertapenem	≤0.25, S	4, R	≥8, R
Gentamicin	8, R	≤1, S	≥16, R
Amikacin	16, R	≤4, S	≤4, S
Ciprofloxacin	≥4, R	≥4, R	≥4, R
Doxycycline	16, NA	≤2, NA	16, NA
Tigecycline	1, R	≤0.25, S	0.5, S
Trimethoprim/sulfamethoxazole	≥8, R	≤0.5, S	≥8, R
Colistin	≤0.25, S	≤0.25, S	≤0.25, S
Antimicrobial resistance genes (ARGs)^b^	*bla*_**CTX-M-15**,_ *bla*_OXA-1,_ *aadA5, aacA4, aac(6′)-Ib-cr, mph(A), catB3, sul1, dfrA17, tet(A), mdf(A)*	*bla*_**OXA-181**,_ *bla*_CMY-42,_ *qnrS1*, *mdf(A)*	*bla*_**NDM-5**,_ *bla*_CMY-2,_ *bla*_TEM-30,_ *aac(3)-IIa, aadA1, aadA2, mdf(A), mph(A), erm(B), floR, sul1, sul2, tet(A), dfrA12, dfrA1*
Virulence factors^b^	*afaA, afaD, chuA, fimH, fimC, gad, hra, iha, iss, kpsE, kpsMII_K5, ompT, papA_F43, sat, senB, traT*	*fimH, gad, terC, yehA, yehB, yehC, yehD*	*csgA, gad, hra, irp2, terC, traT*
Siderophores	*cirA, fepA, fepD, fhuF, fuyA, irp2, iucC, iutA, sitA*	*cirA, fepA, fepD, fhuF*	*cirA, fepA, fepD, fyuA, fhuF, iucC, iutA, sitA*
Bacteriocins	*cbrA, cvpA, tolR*	*cbrA, Colicin, cvp, tolR*	*cib, cvpA, tolR*
Bacteriocins immunity	bacteriocin immunity protein Colicin E7 immunity protein, Colicin transporter	None	Colicin 1B immunity protein
Plasmids^b^	P1: FII/FIB/FIA/Col156 (173Kb)	P1: IncX3 (51Kb); P2: IncI1 (47Kb)	P1: IncI1 (115Kb); P2: IncFII (71Kb); P3: IncFII/FIA/FIB (99Kb)
Sequence type (ST)^b^	ST131	ST410	ST167
Phylogenetic group^c^	B2	A	A
Susceptibility to *INTESTI bacteriophage* cocktail^d^	++	R	R

R, resistant; S, susceptible; NA, not available. ++, susceptible (opaque lysis).

^**a**^ MIC values interpreted according to the EUCAST 2023 criteria (version 13.0; https://www.eucast.org/clinicalbreakpoints/).

^**b**^ Based on the whole-genome sequencing (WGS) and interpreted according to the Genomic Center Epidemiology database (https://www.genomicepidemiology.org/) or the output of Geneious software. The main *bla* genes are indicated in bold.

^**c**^ According to (Clermont et al., 2000).

^**d**^ Susceptibility to the *INTESTI* bacteriophage cocktail was determined by implementing the double layer agar (DLA) method.

^**e**^ This assembly was repeated in the present work and deposited as BioSample SAMN38456712.

### 2.2 Colonization experiments

**Figure 1:**
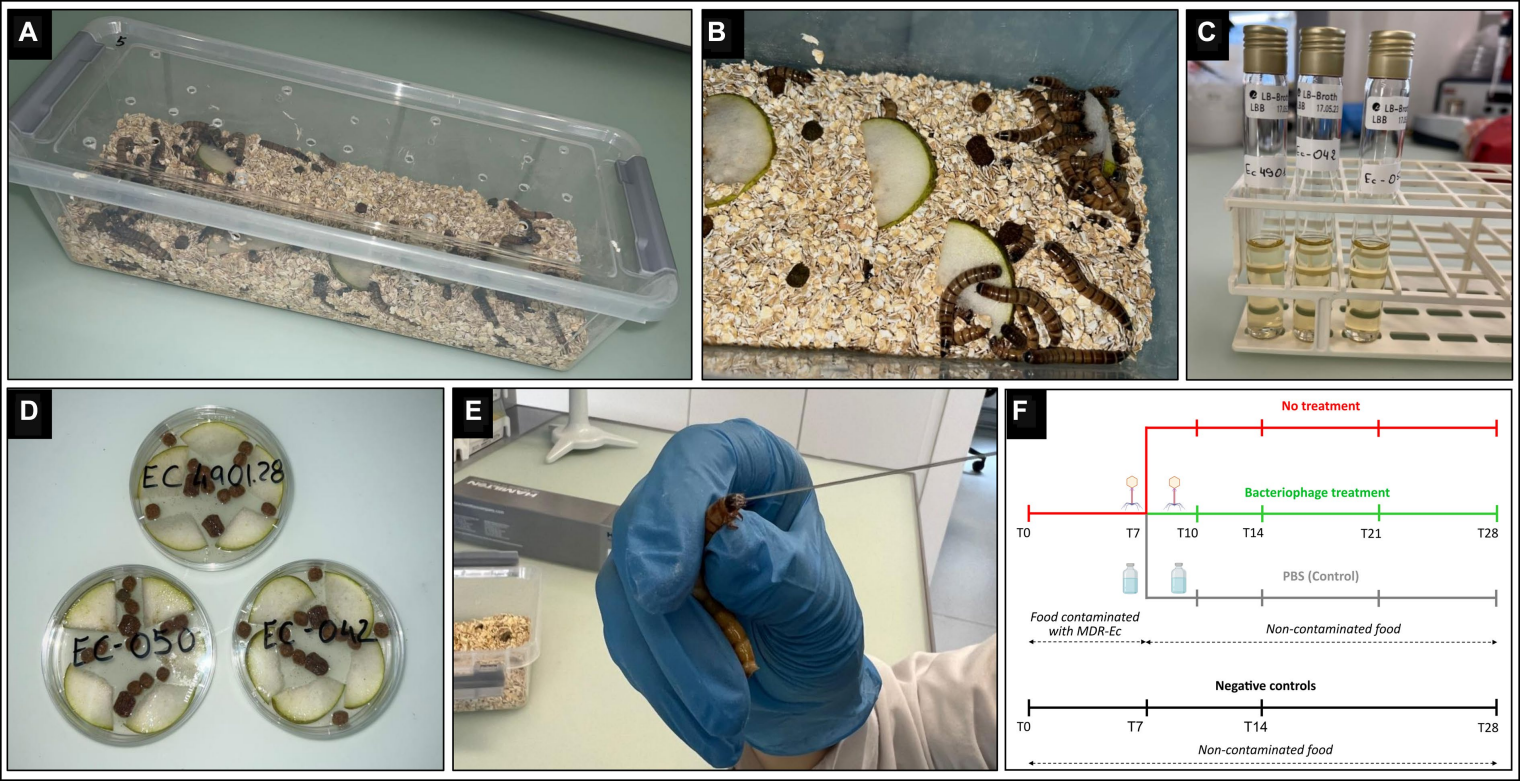
*Zophobas morio* larvae rearing and colonization with multidrug-resistant *Escherichia coli* (MDR-*Ec*) strains. **(A)** Plastic container used as a cage for *Z. morio* larvae. **(B)** The cage has: holes for air exchange, bedding oat substrate, fresh pear pieces, and dry food for cats. **(C)** Tube containing 10 mL of Luria-Bertani (LB) broth where the *E. coli* has grown (overnight incubation). **(D)** Petri dish containing the contaminated food after adding the overnight LB broth; dishes are incubated for 1 h and then food is distributed over the oat substrate. **(E)** Force-feeding: larvae are kept in a fixed position, a small amount of pressure is applied, and the 26 s-gauge needle is inserted into the mouth for injection of 10 μl of *INTESTI bacteriophage* cocktail. **(F)** Study design: timeline showing the timepoints (T, days) for every larvae’s study group.

*Zophobas morio* larvae (box of 40 g, ~60–80 larvae; BUGS-International GmbH) were purchased on different occasions from a Swiss pet store (QUALIPET) and kept in polypropylene containers (33x8x11 cm) at room temperature (23 ± 1°C) with regular day and night light conditions. Larvae were fed daily with slices of fresh pears (rinsed three times with distilled water and then surface disinfected with 70% ethanol) and dry food for cats (~20 g and ~ 3 g, respectively / container) mixed in a substrate of oat flakes (height, ~1 cm). This diet is fully compatible with the physiology of *Z. morio* larvae (Rumbos and Athanassiou, 2021). Moreover, it is commonly used by professionals to bread larvae used for feeding reptiles in captivity (data not shown). Adult larvae (700 ± 50 mg and 45 ± 5 mm in length) were used for all experiments after 2–3 days of acclimatization (Figure 1A).

Notably, direct culture-based analyses on MacConkey II and Columbia sheep blood agar plates (Becton-Dickinson) revealed that disinfected pears were contaminated with *Bacillus cereus* [~3 × 10^1^ colony forming units (CFU)/mL], whereas dry food contained *Moraxella osloensis* and *Micrococcus luteus* (~2–5 × 10^2^ CFU/mL); the oat substrate was constantly negative. Moreover, the three types of food were regularly screened for the presence of extended-spectrum cephalosporin-resistant (ESC-R)-*Ent* using an overnight broth enrichment and selective agar plates as previously done (Endimiani et al., 2020; Campos-Madueno et al., 2023a). As a result, no food used throughout experiments resulted contaminated with ESC-R-*Ent* (data not shown).

To induce gut colonization with the MDR-*Ec*, larvae (at least *n* = 48) were fed for 1 week [from day 0 (T0) to day 7 (T7)] with food contaminated with the specific strain (Figures 1B,F). In particular, colonies grown on a MacConkey II plate were incubated overnight with 10 mL Luria-Bertani (LB) broth (Thermo Fisher Scientific) (Figure 1C). After that, the overall broth was poured into a sterile Petri dish containing slices of pears and dry food, and then further incubated for 1 h at 36 ± 1°C before administration to larvae (Figures 1B,D). Notably, bacterial concentration in the overnight LB broth was ~10^9^ CFU/mL for each of the three tested MDR-*Ec* strains (colony count performed; data not shown).

After T7, larvae were transferred to a new clean cage with a fresh oat substrate where they received non-contaminated food for the next 21 days. For the overall 28 days, the administered food (from T0 to T7 contaminated and from T7 to T28 non-contaminated) was removed every day using sterile tweezers and replaced with a fresh one (Figure 1F).

Colonization experiments were replicated at least 4 times for each of the three MDR-*Ec* strains tested (Supplementary Table S1). Finally, 4 experiments where larvae received only non-contaminated food from T0 to T28 were also performed as negative controls (Neg-Cs; Figure 1F).

### 2.3 Treatment with bacteriophages and controls

The *INTESTI* cocktail was used to test its potential to decolonize larvae from the MDR-*Ec* strains. This cocktail is a sterile-filtrate phage lysate [~10^5–6^ plaque forming unit (PFU)/mL] of *E. coli*, *Shigella* spp., *Salmonella* spp., *Proteus vulgaris*/*mirabilis*, *Pseudomonas aeruginosa*, *Staphylococcus* spp., and *Enterococcus* spp. strains. It was fully characterized with a metagenomic approach (Zschach et al., 2015).

Larvae receiving bacteriophages followed the same protocol as the colonization experiments (see above). However, a sub-group of larvae (at least *n* = 16) received 10 μL of *INTESTI* cocktail administered *per os* by force-feeding on both T7 and T10 using a blunt 26 s-gauge needle connected to a 250 μL Gastight syringe (Hamilton) (Figures 1E,F). For each MDR-*Ec* tested, force-feeding with bacteriophages was replicated in 3 experiments.

As commonly done (e.g., Zhang et al., 2019), colonized larvae were also subjected to 2 further force-feeding experiments in which on both T7 and T10 they received 10 μL of sterile 1X Dulbecco phosphate-buffered saline (1X dPBS; Biochrom GmbH) used as control (Figure 1F; Supplementary Table S1). Notably, larvae were inspected for the first 30 min after any force-feeding and, in case of vomiting, they were discharged and replaced by new ones.

### 2.4 Larvae processing and MDR-*Ec* detection

For each experiment, 4 random larvae were simultaneously sampled at 8 specific time points: T0 (before administering food contaminated with the MDR-*Ec*), T2, T4, T7 and T10 (both before the corresponding force-feeding with bacteriophages or 1X dPBS, if any), T14, T21, and T28. For simplicity, samples taken during experiments with the administration of bacteriophages will be hereafter referred to as T10+, T14+, T21+, and T28+ (Figure 1F).

The 4 larvae taken at each time point were placed inside a 50 mL polypropylene tube (TPP Techno Plastic Products AG) and euthanized by placing them at −20°C for 1 h. After that, their exoskeleton was disinfected by adding 20 mL of 70% ethanol for 3 h at 4°C. The Precellys Evolution Touch tissue homogenizer apparatus (Bertin Technologies) was then implemented to homogenize the 4 disinfected larvae. In particular, two 7 mL high-impact bead beating tubes containing 1.4 mm ceramic beads (Labgene Scientific SA) plus 2 mL of sterile 1X dPBS were loaded with 2 larvae each. Tubes were processed as follows: 20 s/5500 rpm, break 30 s, and 20 s/5500 rpm. Then, the 2 homogenized liquid samples were pooled into a single 15 mL conical tube (Sarstedt AG & Co.) for a final volume of ~3–4 mL.

Final samples were properly diluted in sterile 1X dPBS and aliquots of 100 μL were plated on selective ChromID® ESBL agar plates (bioMérieux), followed by an overnight incubation at 36 ± 1°C in ambient air to detect ESC-R Gram-negatives. After bacterial species identification (see below), colony count (CFU/mL) for the specific MDR-*Ec* strain (ESC-R due to the production of ESBLs and/or carbapenemases) used to colonize the larvae was performed for all the 8 time points. Notably, colony count results were expressed as CFU/mL consistently with similar studies (e.g., Cools et al., 2019). All homogenized samples were stored at −80°C in 2 mL cryogenic Cryo.s tubes (Greiner Bio-One) supplemented with 20% glycerol.

### 2.5 Characterization of MDR-*Ec* recovered from larvae

Colonies grown on ChromID® ESBL agar plates (i.e., ESC-R) were identified at the species level using the matrix-assisted laser desorption/ionization time-of-flight mass spectrometry (MALDI-TOF MS; Brucker).

Six ESC-R-*Ec* strains obtained from different experiments at T28/T28+ (or T21/T21+ if no bacterial growth at T28/T28+) also underwent antimicrobial susceptibility tests (ASTs), phenotypic assay for the *INTESTI bacteriophage* cocktail, whole-genome sequencing (WGS) and single nucleotide variant (SNV) analyses. All results were compared for consistency with the original MDR-*Ec* strains (Table 1) administered from T0 to T7 with the contaminated food.

ASTs were performed using the MIC microdilution Sensititre GNX2F panels (Thermo Fisher Scientific) and interpreted according to the 2023 European Committee on Antimicrobial Susceptibility Testing (EUCAST) breakpoints for *Ent* (v13.0).^1^ Susceptibility to bacteriophages was evaluated using the DLA method (Clockie and Kropinski, 2009).

WGS was achieved using both short- and long-read sequencing. Short-read sequencing was conducted with the Illumina NovaSeq 6,000 platform applying the NEBNext® Ultra™ II DNA library prep kit (2 × 150 bp paired-end reads) by Eurofins Genomics (Ebersberg, Germany), while for long-reads the MinION sequencer (Oxford Nanopore Technologies) was implemented using a rapid barcoding library prep (SQK-RBK004) and FLO-MIN 106D R9.4.1 flow cells. Sequencing adaptors were removed from both short- and long-reads using Trimmomatic v0.36 and Porechop v0.2.3, respectively. Complete and circular genomes were generated using Unicycler v0.4.8^2^ using the hybrid pipeline as previously performed (Campos-Madueno et al., 2020, 2021, 2022). Results were interpreted using the tools of the Center for Genomic Epidemiology,^3^ such as ResFinder v4.1, VirulenceFinder v2.0, PlasmidFinder v2.1, and MLST (all with default parameters).

For the SNV analyses, individual chromosomal and plasmid sequences from every strain were used to generate core-genome alignments using Parsnp v1.7.4^4^ as previously described (Bernasconi et al., 2020; Campos-Madueno et al., 2022; Moser et al., 2022). Each chromosomal and plasmid sequences of the original MDR-*Ec* strains were used as a reference to the recovered ESC-R-*Ec* for core-genome alignment. The recombination filtration (−x parameter) was used and the rest of the parameters were set as default. Alignment coverage of the core-genome was determined automatically with Parsnp. SNV sequences were extracted from the Variant Call Format (VCF) output using Harvest-Tools v1.2.^5^ Only high quality SNVs (PASS) were considered.

### 2.6 Viral population dynamics

The DLA method was also implemented for bacteriophage enumeration (titration) in the homogenized larvae tissues as previously done (Bernasconi et al., 2020). In particular, samples from larvae treated with bacteriophages (250 μL of tissues diluted 1:1 in 1X dPBS) were filtrated using a 0.22 μm pore size PES sterile syringe (Carl Roth Gmbh). The titration was performed for the following time points; T7 (null control), T10+, T14+, T21+, and T28 + .

Briefly, 1.5% Brain Heart Infusion (BHI; Becton-Dickinson) agar was prepared and distributed as a first layer in a sterile Petri dish. Then, 100 μL of the filtrated tissues were added to a 15 mL tube containing 1 mL of BHI broth and incubated for 5 min at room temperature. Subsequently, 100 μL of the previously characterized phage susceptible *E. coli* strain 56-M3-*Ec* (++++; confluent lysis: complete clearing) grown overnight in BHI broth (Bernasconi et al., 2017) plus 4 mL of 0.6% of BHI agar were added to the 15 mL tube and then distributed on top of the first agar layer. Upon an overnight incubation at 36 ± 1°C, confluent lysis plaques on the agar plates were counted for the viral titer (PFU/mL).

### 2.7 Microbiota analysis

For each MDR-*Ec* strain tested, microbiota was analyzed in three different experiments (named A, B, C for simplicity; Supplementary Table S1) where, at T7, larvae were split in two groups: non-treated larvae (samples analyzed: T0, T7, T14, and T28) and larvae receiving bacteriophages (samples analyzed: T14+ and T28+). Finally, microbiota was also analyzed at T0, T7, T14, and T28 for larvae not receiving neither contaminated food nor bacteriophages (i.e., the 4 Neg-Cs; see above).

DNA from the homogenized samples was extracted using the QIAamp PowerFecal Pro DNA Kit (Qiagen). Total gDNA was purified using CleanNA CleanNGS purification beads (Labgene) and resuspended in 10 mM Tris–HCl buffer at pH 8.0 (Sigma-Aldrich). DNA quantification and purity were determined using the NanoDrop™ One/OneC Microvolume UV–Vis spectrophotometer (Thermo Fisher Scientific) and Qubit™ 3.0 Fluorometer (Invitrogen).

Larvae microbiota characterization was achieved through 16S rRNA amplicon sequencing. gDNA extracts were sent for Illumina sequencing to Microsynth AG.^6^ For library preparation, the 16S rRNA V4 region was subjected to Nextera two-steps PCR amplification using the 515F Parada primer (GTGYCAGCMGCCGCGGTAA), and 806R Apprill primer (GGACTACNVGGGTWTCTAAT) (Walters et al., 2016). Sequencing data were generated as adaptor trimmed, demultiplexed and quality checked raw reads in fastq format. Notably, sequence counts of all samples were above 23,000 reads. Identification of the Amplicon Sequence Variants (ASVs) was generated using DADA2 v1.26.0^7^ R package. Taxonomy was assigned using the SILVA-based (v138.1) bacterial reference alignment. Microbial community analysis was performed using the phyloseq v1.38.0^8^ package for R v4.4.2 (McMurdie and Holmes, 2013). The abundance of bacterial taxonomic composition was obtained at the genus level using the DADA2 pipeline and the mirlyn v1.4.0 R package (Moor et al., 2021).

### 2.8 Microbiota diversity analyses

Diversity indexes were determined based on the entire ASV data obtained. The output generated by the DADA2 pipeline was imported as a phyloseq object in R for diversity analyses (McMurdie and Holmes, 2013).

The alpha diversity was analyzed obtaining both richness and the Shannon diversity index (SDI) (Kim et al., 2017). Before conducting within samples comparison, the phyloseq object was normalized using the TMM method (Trimmed Mean of M-value) from the edgeR v3.36.0 R package. Beta diversity was then estimated using the Bray–Curtis dissimilarity index (BCDI) using the vegan package v2.6.4^9^ and the phyloseq package, while the compositional differences between the samples were tested using the permutational analysis of variance (PERMANOVA).

### 2.9 Statistical analysis

All statistical analyses were performed using GraphPad Prism version 9.4.0 for Windows (GraphPad Software). CFU/mL count data were analyzed starting from T10/T10+ to assess differences in bacterial growth rates between the three groups (i.e., larvae not receiving bacteriophages, larvae receiving bacteriophages, and larvae receiving 1X dPBS) using the two-tailed F test of variance.

Microbiota beta diversity of BCDI mean values for (i) Neg-C experiments #1, #2, #3 and #4, and (ii) experiments A, B and C for the three tested MDR-*Ec* strains, were, respectively, statistically compared using two-way ANOVA, followed by Tukey *post hoc* test. A *p-*value <0.05 was considered to be statistically significant.

### 2.10 Data availability

All 16S rRNA gene sequenced samples have been deposited in GenBank under BioProject accession number PRJNA992250. The re-assembled 4901.28_2 strain is deposited under PRJNA551948 BioProject, BioSample ID SAMN38456712. Whole-genome sequences of the 6 MDR-*Ec* strains are deposited under BioProject accession number PRJNA1045999.

## 3 Results

All crude results of each single *in vivo* experiment to determine MDR-*Ec* colonization load (CFU/mL), effect of treatment with bacteriophages and viral titration (PFU/mL) in homogenized larvae samples are depicted in Supplementary Table S1.

### 3.1 Establishing the gut colonization with MDR-*Ec* strains

**Figure 2:**
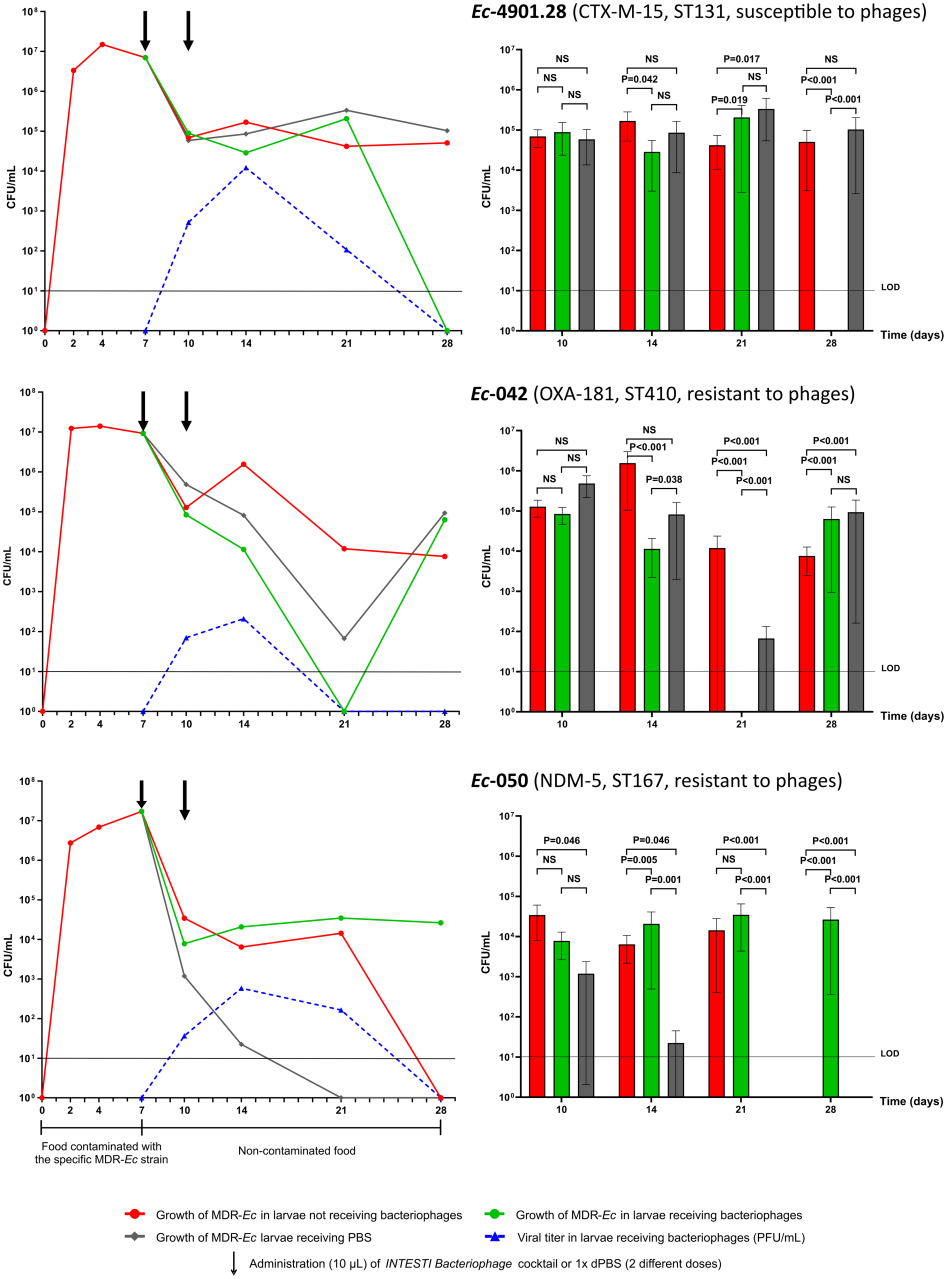
Results of *Z. morio* larvae intestinal colonization with the three multidrug-resistant *E. coli* (MDR-*Ec*) strains: *Ec-*4901.28, *Ec*-042 and *Ec*-050. **(Left)** Growth curves presented as mean of colony forming unit (CFU)/mL [plaque forming unit (PFU)/mL applies only for viral titer]. Black arrows indicate the two time points when bacteriophages or 1X dPBS doses were administered. **(Right)** Statistical investigation of the growth curves. Box plots summarize data presented as mean of CFU/mL, error bars represent standard error of the mean and *p*-values are calculated using *F* test for variances. NS, not significant (i.e., *p* > 0.050). LOD, limit of detection. The graphs were generated with GraphPad Prism 9 on data from all experiments. See Supplementary Table S1 for results of each specific experiment.

A summary (mean) of the growth curve experiments for larvae fed with the three MDR-*Ec* strains is shown in Figure 2 (left panel). Notably, during all of these colonization experiments none of the *Z. morio* larvae died.

After administering the food contaminated with the ST131 *Ec*-4901.28 strain, *Z. morio* larvae became rapidly colonized with a high bacterial load (T7 = 6.95×10^6^ CFU/mL). The removal of contaminated food on T7 induced a drop in bacterial count (T10 = 6.91 × 10^4^ CFU/mL), but this value remained constant in the larvae until the end of experiments (T28 = 5.07 × 10^4^ CFU/mL). Notably, for this specific MDR-*Ec* strain, experiment #6 was extended to 35 days, with the bacterial count still being 1.19×10^4^ CFU/mL (Supplementary Table S1).

The ST410 *Ec*-042 strain, showed a similar capacity to colonize *Z. morio* larvae (T7 = 9.22×10^6^ CFU/mL) as *Ec*-4901.28, although at the end of experiments bacterial load was slightly lower (T28 = 7.60×10^3^ CFU/mL). In contrast, the ST167 *Ec*-050 strain displayed a different colonization behavior. Specifically, feeding with contaminated food generated a colonization load of 1.71 × 10^7^ CFU/mL on T7, but then, bacterial count in larvae rapidly declined to 0 CFU/mL on T28.

### 3.2 Impact of bacteriophages treatment on MDR-*Ec* colonization

Colony counts for homogenized larvae that underwent force-feeding with the *INTESTI bacteriophage* cocktail, together with the corresponding viral titers, are summarized in Figure 2 (left panel). Statistical analysis of the overall growth curves is also shown in Figure 2 (right panel). Notably, ~15% of force-fed larvae were discharged because of vomiting, while none of those remaining in the experiments died by T28+.

For the *INTESTI*-susceptible *Ec*-4901.28 strain, treatment with bacteriophages induced a decrease in the MDR-*Ec* load that ended up with CFU/mL count lower than the limit of detection (LOD) on T28+. This final effect was consistent with a high viral titer recorded at T14+ (1.22×10^4^ PFU/mL); such titer decreased to 0 on T28+, in parallel to the disappearance of *Ec*-4901.28. Notably, treatment with 1X dPBS (control) did not have any effect (T28 = 1.03×10^5^ CFU/mL), as shown by the resultant bacterial growth curve that was similar to the one obtained for larvae not receiving the bacteriophage cocktail. Statistical analysis showed that the *Ec*-4901.28 growth curves for larvae receiving and not receiving bacteriophages displayed a significant difference to each other from T14/T14+ (*p* = 0.042) to T28/T28+ (*p* < 0.001). In contrast, at T28, growth curves for non-treated larvae and those receiving 1X dPBS were not statistically different.

Bacteriophages administration decolonized *Z. morio* larvae from the *INTESTI*-resistant *Ec*-042 on T21+ (CFU/mL count <LOD). This effect was in line with the viral titer curve at T14+ (2.10×10^2^ PFU/mL) and T21+ (0 PFU/mL). However, after T21+, *Ec*-042 started to regrow and larvae returned colonized with this pathogen (T28 + =6.33 × 10^4^ CFU/mL). Analysis of variance indicated that the *Ec*-042 growth curves of larvae treated and non-treated with bacteriophages were significantly different from T14/T14+ to T28/T28+ (*p* < 0.001). Of note, the 1X dPBS treatment curve resembled the curve referring to the bacteriophage’s treatment more than to the one obtained for untreated larvae.

With regard to the *INTESTI*-resistant *Ec*-050 strain, the double dose of bacteriophages did not have an effect on the bacterial load of larvae (T7 = 1.71×10^7^ CFU/mL to T14+ =2.07×10^4^ CFU/mL), although the viral titer reached its peak on T14+ (5.87×10^2^ PFU/mL). In contrast, administration of 1X dPBS led to a decrease of the CFU/mL count of *Ec*-050 under the LOD within T21. However, additional growth curve experiments indicated that 1X dPBS had no effect on the *in vitro* growth of *Ec*-050 (Supplementary Table S2, including methods implemented). Finally, statistical analyses showed that the three bacterial growth curves for this MDR-*Ec* strain were significantly different to each other from T14/T14+ to T28/T28+ (e.g., T28/T28+: all *p* < 0.001).

### 3.3 Stability of MDR-*Ec* strains recovered during experiments

The six MDR-*Ec* strains that underwent full characterization were obtained as follows: experiment #9 (T28) and #11 (T21+) for *Ec*-4901.28, experiment #2 (T28) and #6 (T28+) for *Ec*-042, and experiment #2 (T21) and #7 (T28+) for *Ec*-050.

Antibiotic/bacteriophage susceptibility profiles and genetic backgrounds (e.g., ARGs, plasmids, ST) of the above six strains were consistent with the corresponding original MDR-*Ec* administered with the contaminated food (Table 1). Moreover, the chromosomal SNVs recorded for *Ec*-4901.28, *Ec*-042 and *Ec*-050 at the end of *in vivo* experiments were: T28= 14 and T21+ =29 SNVs; T28 = 5 and T28+ =26 SNVs; and T21= 13 and T28+ =16 SNVs, respectively (Supplementary Table S3).

### 3.4 Original microbiota composition and diversity

**Figure 3:**
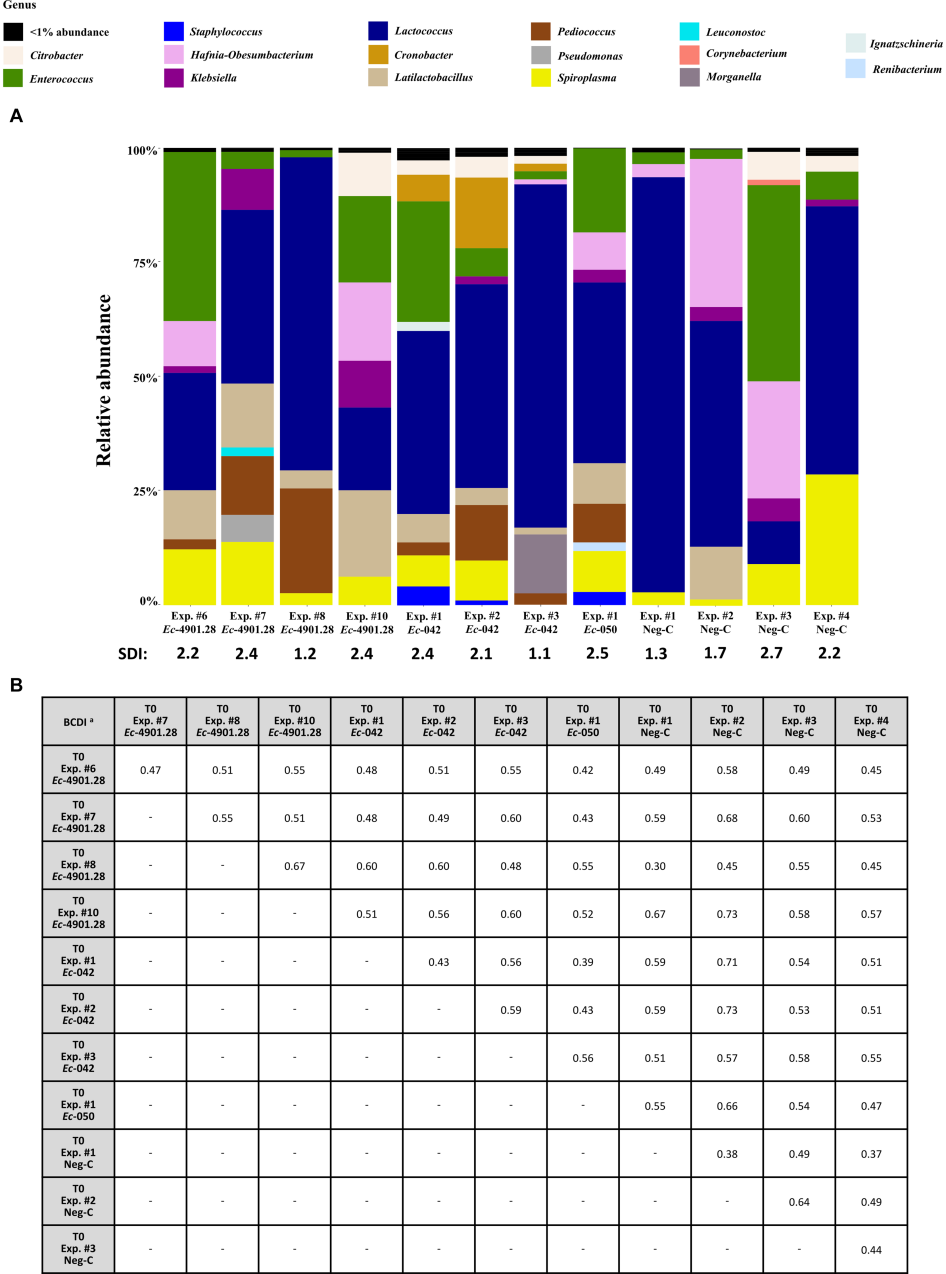
Analysis of the 16S rRNA amplicon sequencing of the homogenized *Z. morio* larvae at all T0 experiments (before colonization experiments, if any). **(A)** The graphic summarizes the microbiota analysis on genus level of larvae at T0 from different experiments, considering the top 30 Amplicon Sequence Variants (ASVs). **(B)** Beta diversity analysis based on BCDI values were obtained comparing the microbial community compositions between the samples (PERMANOVA analysis). BCDI, Bray–Curtis dissimilarity index; Neg-C, negative control; SDI, Shannon diversity index; Exp., experiment. ^**a**^Dissimilarity values were obtained comparing the BCDI between the two samples (range 0 to 1, corresponding to 0 to 100%).

Relative abundance of bacterial genera and SDI of larvae’s microbiota at T0 are described in Figure 3A and Supplementary File S1.

Microbiota patterns for the 12 T0 samples (including the 4 Neg-Cs) appeared heterogeneous, with SDIs ranging between 1.1 and 2.7. Furthermore, comparison to each other (beta diversity) demonstrated BCDIs ≥30% and up to 73% (Figure 3B). Nevertheless, these different initial microbiota patterns had a frequent presence of the following bacterial genera (relative abundance, range): *Lactococcus* (7–88%), *Enterococcus* (2–36%), *Spiroplasma* (1–22%), *Hafnia-Obesumbacterium* (0–31%), *Pediococcus* (0–22%), *Cronobacter* (0–13%), *Latilactobacillus* (0–11%), *Klebsiella* (0–7%), and *Citrobacter* (0–5%) spp.

### 3.5 Microbiota dynamics for negative controls (Neg-Cs)

**Figure 4:**
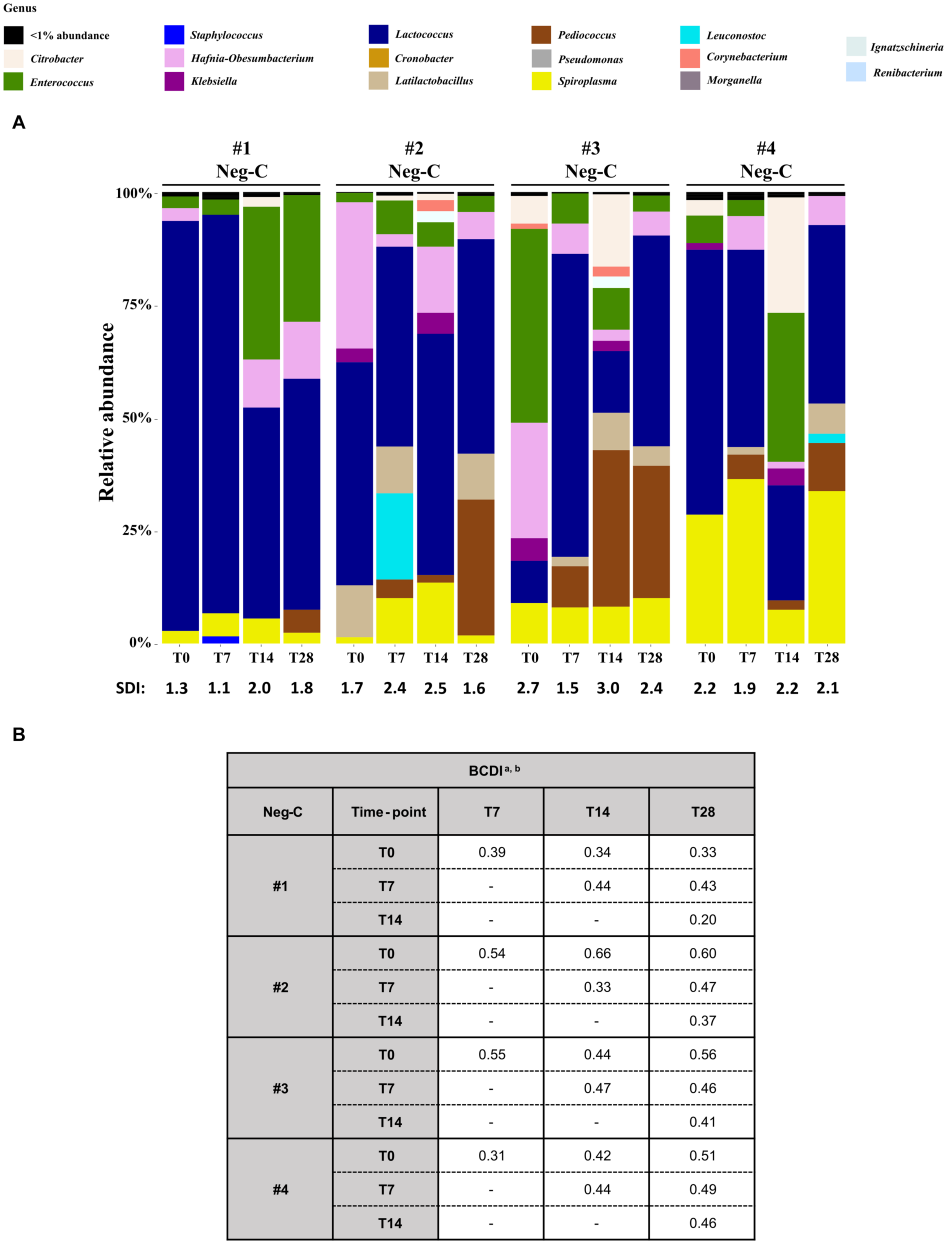
Analysis of the 16S rRNA amplicon sequencing of the homogenized *Z. morio* larvae Neg-Cs (without challenging with MDR-*Ec* and/or bacteriophages). **(A)** The graphic summarizes the microbiota analysis on genus level of larvae at T0 from different experiments, considering the top 30 Amplicon Sequence Variants (ASVs). **(B)** Beta diversity analysis based on Bray–Curtis dissimilarity index (BCDI). Values were obtained comparing the microbial community compositions between the samples. BCDI, Bray–Curtis dissimilarity index; Neg-C, negative control; SDI, Shannon diversity index. ^**a**^Dissimilarity values were obtained comparing the BCDI between the two samples (range 0–1, corresponding to 0–100%). See Supplementary Figure S1 for median and interquartile ranges. ^**b**^Statistical analysis was performed using the mean of BCDI for experiments #1, #2, #3, and #4 together. As a result, all *p*-values were not significant (data not shown).

The larvae microbiota dynamics during the 28 days in the 4 Neg-Cs are depicted in Figure 4, Supplementary Figure S1, and Supplementary File S1.

Considering the relative abundance, two genera were constantly present from T0 to T28: *Lactococcus* (rates up to 88 and 46%, respectively) and *Spiroplasma* (rates up to 21.5 and 33.4%, respectively). Moreover, the SDI ranges for Neg-Cs #1, #2, #3 and #4 were 1.1–2.0, 1.6–2.5, 1.5–3.0 and 1.9–2.2, respectively (Figure 4A). Although the BCDIs between samples of the same experiment were ≥ 20% and up to 66%, statistical comparison showed that they were not significantly different (Figure 4B).

### 3.6 Microbiota dynamics of challenged larvae

**Figure 5:**
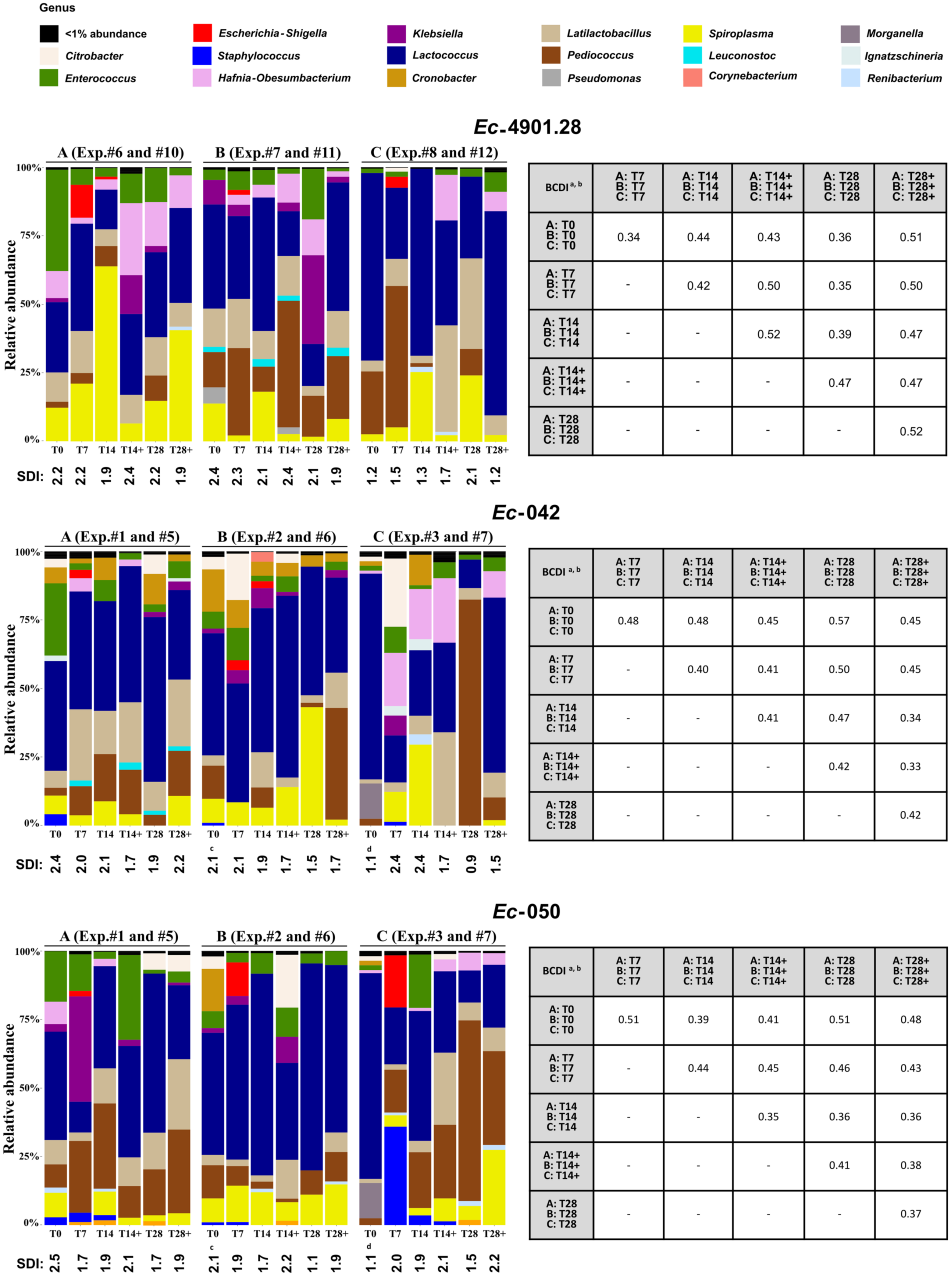
Analysis of the 16S rRNA amplicon sequencing of the larvae homogenized samples. **(Left)** For each MDR-*Ec* strain tested, we analyzed the microbiota from experiments A, B, and C without use of bacteriophages (samples analyzed: T0, T7, T14, and T28) and where bacteriophages were administered (samples analyzed: T14+ and T28+); see Supplementary Table S1 for specific experiment #. The graphic summarizes the microbiota analysis on genus level considering the top 30 Amplicon Sequence Variants (ASVs). SDI, Shannon diversity index values for every sample. **(Right)** Beta diversity analysis based on the BCDI. Mean values were obtained comparing the microbial community composition between the samples. Diversity analysis was calculated based on the total microbial abundance after TMM normalization. BCDI, Bray–Curtis dissimilarity index; Exp., experiment. ^a^Dissimilarity values were obtained comparing the mean of BCDI for A, B, and C at different time points (range 0–1, corresponding to 0–100%). See Supplementary Figure S2 for median and interquartile range. ^b^Statistical analysis was performed using the mean of A, B, and C at different time points. As a result, all *p*-values were not significant (data not shown). ^c^T0 was the same for these experiments. ^d^T0 was the same for these experiments.

The dynamics of relative genus abundance and SDI recorded in three different experiments (A, B, and C) performed with or without administering bacteriophages are depicted in Figure 5 (left panel), Supplementary Figure S2, and Supplementary File S1.

Overall, several general aspects can be noted. First, the *Escherichia*-*Shigella* genera appeared in larvae only at T7 (relative abundance range: 1–20%), but it mostly disappeared (<1% abundance) at T14/T14+, regardless of the administration of bacteriophages. Second, *Enterococcus* spp. tended to decrease from the beginning of colonization experiments and, irrespective of the use of *INTESTI* cocktail, its relative abundance at T28/T28+ was usually lower than at the corresponding T0. Third, when *Citrobacter* spp. was part of the microbiota at T0, the use of bacteriophages was related to its elimination at T28+; this phenomenon was only partially true when *Klebsiella* spp. was naturally present in the microbiota of larvae. Last, in most of the experiments, community diversity (SDI) of individual samples at T28/T28+ was slightly lower than the initial microbial diversity at T0; although a statistical difference between the BCDIs was not recorded (Figure 5, right panel).

Notably, in the experiments where the *INTESTI*-susceptible strain *Ec*-4901.28 was used, the bacterial diversity after the administration of bacteriophages was always lower than when no treatment was implemented (e.g., in experiment A: SDI = 1.9 at T28+, whereas SDI = 2.2 at T28); however, no statistical differences between the BCDIs were observed (Figure 5, right panel).

## 4 Discussion

With the pandemic increase of difficult to treat MDR-*Ec* infections, there is a public health need for *in vivo* models to test novel approaches to decolonize intestinal carriers and to better understand their microbiota dynamics (Bonomo et al., 2018; Peirano and Pitout, 2019; Campos-Madueno et al., 2023b). In this context, animal experiments - especially those with mice - remain essential (Perez et al., 2011; Javaudin et al., 2021; Stercz et al., 2021; Fang et al., 2022). However, to overcome their associated complex ethical aspects, high costs, and limited feasibility (Freires et al., 2017), new alternative *in vivo* models based on the 3Rs strategy should be developed (Sneddon et al., 2017).

One of such replacement strategies is the invertebrate model *Galleria mellonella* (Singkum et al., 2019). Using its larvae, Lange et al. successfully established an oral administration model using commensal bacteria to study innate immune responses (Lange et al., 2019). More recently, Mirza et al. designed a gut colonization model with carbapenemase-producing *Ent* indicating that the use of *E. coli* strains generated high mortality rates in *G. mellonella* larvae (Mirza et al., 2024).

The implementation of *G. mellonella* larvae was also explored in our laboratory to induce gut colonization with MDR-*Ec* strains, but the following critical drawbacks were noted (data not shown): (i) handling the larvae posed a challenge due to their fragile exoskeleton; (ii) larvae refused the oral force-feeding and, when administered, it was detrimental (i.e., rapid and high mortality rates); and (iii) larvae did not eat the diet provided (Singkum et al., 2019). Moreover, *G. mellonella* has been shown to have a less diverse microbiota dominated mostly by *Enterococcus* spp. and, importantly, does not tolerate colonization with *Ent* well, especially with *E. coli* (Allonsius et al., 2019; Mirza et al., 2024). Therefore, we focused our attention to the *Z. morio* larvae which could overcome the above-mentioned downsides due to the physiologic characteristics so far known (Luo et al., 2021; Rumbos and Athanassiou, 2021).

### 4.1 *Zophobas morio* larvae may be persistently colonized with hyperepidemic MDR-*Ec* strains

To establish our new model, larvae were first tested with three clinically and epidemiologically relevant *MDR-Ec* strains that nowadays are frequently responsible for gut colonization in humans and animals (Table 1). Consequently, larvae were rapidly colonized with a high bacterial load of > 10^6^ CFU/mL at T2 and > 10^7^ CFU/mL at T7. Nevertheless, each specific strain had a different colonization behavior after the cessation of contaminated food administration (Figure 2).

The first strain tested (*Ec*-4901.28) was a CTX-M-15 producer of ST131 lineage, being globally the most dominant extra-intestinal pathogenic *E. coli* strain often responsible for community- and hospital-acquired urinary-tract infections and/or bacteremia (Seiffert et al., 2013; Peirano and Pitout, 2019; Pitout and Finn, 2020). The strain was able to persist within the larvae with a stable bacterial load from T10 to T28 (~10^5^ CFU/mL; Figure 2). This phenomenon was also observed in our previous work where we used a bioreactor to simulate the human gut (Bernasconi et al., 2020). Overall, these results can be explained by the molecular features of ST131 that favor its fitness and long-term intestinal colonization capacities (Pitout and Finn, 2020). In fact, *Ec*-4901.28 carries a higher number of virulence factors (VFs) that aid the adherence/invasion of host intestinal epithelial cells, siderophores to scavenge iron, bacteriocins and corresponding immunity proteins that confer an advantage over sensitive bacterial populations (Table 1).

The second MDR-*Ec* strain used (*Ec*-042) was an OXA-181 producer belonging to ST410, a rapidly emerging pandemic clone particularly able to colonize the intestinal tract of humans and animals (Nigg et al., 2019; Moser et al., 2021; Pitout et al., 2024). As recorded for *Ec*-042 (Table 1), the ST410 lineage possesses patterns of VFs (e.g., fimbriae, bacteriocins) very similar to those of ST131 (Roer et al., 2018; Feng et al., 2019; Pitout et al., 2024). Therefore, it is not surprising that *Ec*-042 also rapidly colonized *Z. morio* larvae and persisted with a high bacterial load until T28 (~10^4^ CFU/mL; Figure 2).

In contrast to the above two MDR-*Ec*, *Ec*-050 expressed the weakest colonization effect on larvae as no colonies were present after T21 (Figure 2). This strain produces the NDM-5 carbapenemase and belongs to another worldwide emerging high-risk clone (ST167) that spreads in human and non-human settings (Endimiani et al., 2020; Schmidt et al., 2020; Linkevicius et al., 2023). As for *Ec*-4901.28 and *Ec*-042, *Ec*-050 carried numerous genes encoding VFs (Table 1). Therefore, we are unable to clearly explain its inability to persistently colonize larvae. Nevertheless, based on the findings of larvae receiving bacteriophages, a hypothesis can be raised (see below).

### 4.2 Bacteriophages can decolonize larvae when active against the MDR-*Ec* strain

As a proof of concept to test new strategies to decolonize intestinal carriers of MDR-*Ec* (Campos-Madueno et al., 2023b), our colonized larvae were challenged with two doses of *INTESTI bacteriophage* cocktail at T7 and T10 (Figure 2).

Numerous studies have shown that bacteriophages are highly active *in vivo* against infections due to MDR-*Ec*. In contrast, data regarding the effect of bacteriophages against the intestinal colonization due to MDR-*Ent* in *in vivo* models are scarce (Lin et al., 2017; Campos-Madueno et al., 2023b). Using four lytic phages, Javaudin et al. were unable to decolonize mice carrying ESBL- or OXA-48-producing *E.coli* strains (Javaudin et al., 2021). Fang et al. showed that administration of two lytic phages to mice colonized with a carbapenem-resistant *K. pneumoniae* strain generated phage-resistant mutants (Fang et al., 2022). In the study of Mirza et al., administration of two bacteriophages decreased the CFU count of a carbapenemase-producing *K. pneumoniae* colonizing the gut of *G. mellonella*, but the difference was not statistically significant (Mirza et al., 2024).

In our study, larvae were decolonized from the *INTESTI*-susceptible strain *Ec*-4901.28 within T28+ (*p* < 0.001), and no phage-resistant mutants were detected (Figure 2). This latter phenomenon was unexpected, since in our past bioreactor experiments with *Ec*-4901.28 mutants were sometimes isolated, though their mechanism of resistance was not elucidated by WGS analyses (Bernasconi et al., 2020). We therefore speculate that the combination of the multiple lytic phages into the *INTESTI* cocktail, together with the host immune response, reduced the chances of developing resistance against bacteriophages (Lin et al., 2017).

On the other hand, administration of the *INTESTI* cocktail was ineffective for *Z. morio* larvae colonized with phage-resistant strains (Figure 2). In the case of *Ec*-042, there was initially a difficult to explain decrease of the pathogen load (less than the LOD) followed by a rapid regrow (~10^5^ CFU/mL at T28+). More interestingly, *Ec*-050 was not at all affected by the bacteriophages activity and maintained a load significantly higher than during experiments with larvae not receiving treatment (T28 *vs*. T28+: p < 0.001). To explain this interesting data, we hypothesize that there are specific bacterial competitors of *Ec*-050 in the intestinal tract of *Z. morio* larvae that protect against its colonization (a phenomenon known as “colonization resistance”) (Caballero-Flores et al., 2023). Since the *INTESTI* cocktail contains bacteriophages inhibiting multiple species (Zschach et al., 2015) - including the hypothetical competitors - *Ec*-050 could persistently colonize larvae (~10^4–5^ CFU/mL at T28+).

During the experiments with *Ec*-050, we also noted a strong decolonization activity generated by the administration of 1X dPBS (control; Figure 2). This surprising phenomenon did not find an explanation after verifying that *Ec*-050 - as for *Ec*-4901.28 and *Ec*-042 - is not inhibited by 1X dPBS *in vitro* (Supplementary Table S2). Therefore, we speculate that the use of 1X dPBS might: (i) stimulate some species part of the natural larvae microbiota to produce inhibitors (e.g., bacteriocins) against *Ec*-050 (Wang et al., 2024); (ii) affect the characteristics of the gut (e.g., expression of glycoproteins in the epithelium) in an unfavorable way for the *Ec*-050 colonization (Foley et al., 2021); and/or (iii) improve the host immune response against *Ec*-050 (Lange et al., 2019; von Bredow et al., 2023).

### 4.3 Hyperepidemic MDR-*Ec* strains are stable *in vivo*

Recently, we have shown that *Ec*-042 and *Ec*-050 were genetically highly stable after 20 propagation steps on selective agar plates (0 and 0–9 SNVs, respectively) (Moser et al., 2022).

In the present work (Supplementary Table S3), we demonstrated that the three hyperepidemic strains tested behave in a similar way during the *in vivo* experiments with larvae (e.g., 14, 5, and 13 chromosomal SNVs for *Ec*-4901.28, *Ec*-042, and *Ec*-050, respectively), though administration of bacteriophages seems to slightly increase the chance of mutations (e.g., 29, 26, and 16 chromosomal SNVs, respectively). This is consistent with the notion that bacteriophages stress may select for mutants that frequently involve surface receptors (Chevallereau et al., 2022). Overall, our data indicate that the three MDR-*Ec* strains used in our experiments possess stable phenotypic and molecular features that make them very useful for any kind of reproducible *in vivo* experiment.

### 4.4 Purchased *Zophobas morio* larvae possess a rich microbiota

*Zophobas morio* larvae acquired from the pet shop possessed a very diversified gut microbiota, with *Lactococcus*, *Enterococcus* and *Spiroplasma* as dominant and constantly present bacterial genera (Figure 3; Supplementary File S1). This variety may possibly be due to the type of food administered to larvae during their industrial breeding, although the origin and type of these sources are not stated by the provider.^10^ In this context, some authors have reported that restaurant/household/gardening waste, slaughterhouse products and animal manure might be used to breed *Z. morio* larvae (Harsanyi et al., 2020). As a consequence, a high load of human/animal derived bacteria (e.g., *Enterococcus* spp. and *Ent*) could be ingested by the larvae.

We emphasize that the natural richness of bacterial species found in *Z. morio* larvae has no correspondence to other invertebrates (e.g., *G. mellonella* larvae) or genetically modified vertebrates (e.g., gnotobiotic mice) that are frequently used for laboratory experiments (Allonsius et al., 2019; Darnaud et al., 2021). Moreover, the *Z. morio* microbiota recorded at different T0 samples (Figure 3) has a certain stability over time (Figure 4).

### 4.5 Larvae microbiota undergoes non-significant dynamic changes when challenged

When colonization with the MDR-*Ec* strains was induced, larvae microbiota gradually showed a non-significant reduction of the bacterial diversity (SDI) during the 28-day experiments (Figure 5). Since the presence of the colonizing MDR-*Ec* was recorded only at T7 (i.e., *Escherichia*-*Shigella* genus), we speculate that this slight diversity reduction was mainly due to the fixed diet that we administered to larvae rather than the direct effect of the colonizer *per se* (Mason et al., 2020). As anticipated above (see methods), oats, pears and dry food were not contaminated with *Enterococcus* spp. and *Ent*, justifying the reduction of these bacterial species in the gut of *Z. morio* larvae.

As previously observed in humans and animal models (Hong et al., 2016; Febvre et al., 2019), the use of bacteriophages generated in larvae a non-significant reduction in bacterial diversity as for the experiments without treatment; this was independent on the specific MDR-*Ec* strain tested (Figure 5). Moreover, though the microbiota of larvae receiving or not receiving bacteriophages showed to be quite dissimilar (e.g., mean of BCDI at T28+ *vs*. T28 of 37–52%), such patterns were not significantly different. Nevertheless, administration of bacteriophages proved to neutralize *Citrobacter* spp. that by definition should be susceptible to the *INTESTI* cocktail (Zschach et al., 2015). In contrast, *Klebsiella* spp. was not affected due to the lack of lytic phages into the cocktail and some residual *Enterococcus* spp. persisted at T28+ probably due to their phage-resistant profile. Overall, our results confirm that bacteriophages are highly host-specific and have limited effect on the untargeted natural gut bacteria when implemented (Hong et al., 2016; Febvre et al., 2019).

## 5 Conclusion and future prospects

This is the first time that *Z. morio* larvae are implemented as a gut colonization *in vivo* model. In particular, we showed that larvae possess a rich microbiota and can be easily colonized with at least two clinically important global clones of ESBL and/or carbapenemase-producing *E. coli* strains (i.e., ST131 and ST410) via the administration of previously prepared contaminated food (Rinninella et al., 2019; Pitout and Finn, 2020; Luo et al., 2021; Linkevicius et al., 2023; Pitout et al., 2024). Therefore, this new model promises to be a feasible and high-throughput compromise to study novel gut decolonization strategies for MDR-*Ent* (not only *E. coli*) before implementing more accredited models. We underline that it is not the intention of the present *Z. morio* larvae model to replace completely the gold-standard mice model, but just to provide a rapid screening of the recently developed decolonization strategies against MDR bacteria reducing the number of subsequent confirmatory mammalian experiments (Freires et al., 2017).

Nevertheless, this work also indicates that our approach should undergo improvements in the future. Although for an explorative analysis we performed an adequate number of repeated measures (Olsson et al., 2022), it is desirable for them to be increased in the forthcoming experiments, especially those focusing on microbiota dynamics and considering further clinically relevant colonizing species (e.g., *Klebsiella* and *Salmonella* spp.). Moreover, the time required to induce colonization and observe the effect of a decolonization strategy could be shortened by providing contaminated food for only 2–4 days (Figure 2); an orally injected suspension of MDR-*Ent* should also be explored as well. We also speculate that larvae microbiota might be easily adapted and modified to different needs by simply changing the food and its bacterial contamination administered during breeding (Mason et al., 2020). Finally, as already done for *G. mellonella*,^11^ providing genetically stable *Z. morio* larvae (genome sequenced) along with a defined microbiota will be essential to create standardized research grade lines able to deliver more reliable and reproducible gut colonization/decolonization *in vivo* results following the perspectives of the 3R approach.

## Data availability statement

All 16S rRNA gene sequenced samples have been deposited in GenBank under BioProject accession number PRJNA992250. The re-assembled 4901.28_2 strain is deposited under PRJNA551948 BioProject, BioSample ID SAMN38456712. Whole-genome sequences of the 6 MDR-Ec strains are deposited under BioProject accession number PRJNA1045999.

## Ethics

Ethical approval was not required for the studies on animals in accordance with the local legislation and institutional requirements.

## Author contributions

YE: Data curation, Formal analysis, Investigation, Visualization, Writing – original draft, Writing – review & editing. CA: Data curation, Formal analysis, Investigation, Visualization, Writing – original draft, Writing – review & editing. EC-M: Software, Visualization, Writing – original draft, Writing – review & editing, Data curation, Formal analysis, Investigation, Methodology, Resources. AM: Data curation, Formal analysis, Investigation, Methodology, Software, Visualization, Writing – original draft, Writing – review & editing. CK: Data curation, Formal analysis, Investigation, Visualization, Writing – original draft, Writing – review & editing. VP: Supervision, Writing – original draft, Writing – review & editing. MH: Conceptualization, Data curation, Formal analysis, Investigation, Methodology, Software, Supervision, Validation, Visualization, Writing – original draft, Writing – review & editing. AE: Conceptualization, Data curation, Formal analysis, Funding acquisition, Investigation, Methodology, Project administration, Resources, Supervision, Validation, Writing – original draft, Writing – review & editing.

## Footnotes

^1^ https://www.eucast.org/clinical_breakpoints

^2^ https://github.com/rrwick/Unicycler

^3^ https://www.genomicepidemiology.org/

^4^ https://github.com/marbl/parsnp

^5^ https://github.com/marbl/harvest-tools

^6^ www.microsynth.com

^7^ https://github.com/benjjneb/dada2/releases

^8^ https://github.com/joey711/phyloseq

^9^ https://cran.r-project.org/web/packages/vegan/vegan.pdf

^10^ www.bugs-international.com/aufzucht.html

^11^ https://www.impact2amr.com/companies/biosystems-technology/

## References

[ref1] Allonsius C. N. Van Beeck W. De Boeck I. Wittouck S. Lebeer S. (2019). The microbiome of the invertebrate model host *Galleria mellonella* is dominated by *Enterococcus*. Anim. Microbiome 1:7. doi: 10.1186/s42523-019-0010-6, PMID: 33499945 PMC7807499

[ref2] Bernasconi O. J. Campos-Madueno E. I. Dona V. Perreten V. Carattoli A. Endimiani A. (2020). Investigating the use of bacteriophages as a new decolonization strategy for intestinal carriage of CTX-M-15-producing ST131 *Escherichia coli*: an *in vitro* continuous culture system model. J. Glob. Antimicrob. Resist. 22, 664–671. doi: 10.1016/j.jgar.2020.05.018, PMID: 32590187

[ref3] Bernasconi O. J. Dona V. Tinguely R. Endimiani A. (2017). In vitro activity of three commercial bacteriophage cocktails against multidrug-resistant *Escherichia coli* and *Proteus* spp. strains of human and non-human origin. J. Glob. Antimicrob. Resist. 8, 179–185. doi: 10.1016/j.jgar.2016.12.013, PMID: 28232228

[ref4] Bonomo R. A. Burd E. M. Conly J. Limbago B. M. Poirel L. Segre J. A.. (2018). Carbapenemase-producing organisms: A global scourge. Clin. Infect. Dis. 66, 1290–1297. doi: 10.1093/cid/cix893, PMID: 29165604 PMC5884739

[ref5] Caballero-Flores G. Pickard J. M. Nunez G. (2023). Microbiota-mediated colonization resistance: mechanisms and regulation. Nat. Rev. Microbiol. 21, 347–360. doi: 10.1038/s41579-022-00833-7, PMID: 36539611 PMC10249723

[ref6] Campos-Madueno E. I. Aldeia C. Perreten V. Sendi P. Moser A. I. Endimiani A. (2023a). Detection of *bla*_CTX-M_ and *bla*_DHA_ genes in stool samples of healthy people: comparison of culture- and shotgun metagenomic-based approaches. Front. Microbiol. 14:1236208. doi: 10.3389/fmicb.2023.1236208, PMID: 37720151 PMC10501143

[ref7] Campos-Madueno E. I. Bernasconi O. J. Moser A. I. Keller P. M. Luzzaro F. Maffioli C.. (2020). Rapid increase of CTX-M-producing *Shigella sonnei* isolates in Switzerland due to spread of common plasmids and international clones. Antimicrob. Agents Chemother. 64, e01057–20. doi: 10.1128/AAC.01057-20 32718957 PMC7508577

[ref8] Campos-Madueno E. I. Moradi M. Eddoubaji Y. Shahi F. Moradi S. Bernasconi O. J.. (2023b). Intestinal colonization with multidrug-resistant Enterobacterales: screening, epidemiology, clinical impact, and strategies to decolonize carriers. Eur. J. Clin. Microbiol. Infect. Dis. 42, 229–254. doi: 10.1007/s10096-023-04548-2, PMID: 36680641 PMC9899200

[ref9] Campos-Madueno E. I. Moser A. I. Jost G. Maffioli C. Bodmer T. Perreten V.. (2022). Carbapenemase-producing *Klebsiella pneumoniae* strains in Switzerland: human and non-human settings may share high-risk clones. J. Glob. Antimicrob. Resist. 28, 206–215. doi: 10.1016/j.jgar.2022.01.016, PMID: 35085791

[ref10] Campos-Madueno E. I. Moser A. I. Risch M. Bodmer T. Endimiani A. (2021). Exploring the global spread of *Klebsiella grimontii* isolates possessing *bla*_VIM-1_ and *mcr-9*. Antimicrob. Agents Chemother. 65:e0072421. doi: 10.1128/AAC.00724-21, PMID: 34181480 PMC8370233

[ref11] Chevallereau A. Pons B. J. Van Houte S. Westra E. R. (2022). Interactions between bacterial and phage communities in natural environments. Nat. Rev. Microbiol. 20, 49–62. doi: 10.1038/s41579-021-00602-y 34373631

[ref12] Clermont O. Bonacorsi S. Bingen E. (2000). Rapid and simple determination of the *Escherichia coli* phylogenetic group. Appl. Environ. Microbiol. 66, 4555–4558. doi: 10.1128/AEM.66.10.4555-4558.2000, PMID: 11010916 PMC92342

[ref13] Clockie M.R.J. Kropinski A.M. (2009). Bacteriophages: Methods and protocols, vol 1. Isolation, characterization, and interactions. New York: Humana Press.

[ref14] Cools F. Torfs E. Aizawa J. Vanhoutte B. Maes L. Caljon G.. (2019). Optimization and characterization of a *galleria mellonella* larval infection model for virulence studies and the evaluation of therapeutics against *Streptococcus pneumoniae*. Front. Microbiol. 10:311. doi: 10.3389/fmicb.2019.00311, PMID: 30846978 PMC6394149

[ref15] Darnaud M. De Vadder F. Bogeat P. Boucinha L. Bulteau A. L. Bunescu A.. (2021). A standardized gnotobiotic mouse model harboring a minimal 15-member mouse gut microbiota recapitulates SOPF/SPF phenotypes. Nat. Commun. 12:6686. doi: 10.1038/s41467-021-26963-9, PMID: 34795236 PMC8602333

[ref16] Endimiani A. Brilhante M. Bernasconi O. J. Perreten V. Schmidt J. S. Dazio V.. (2020). Employees of Swiss veterinary clinics colonized with epidemic clones of carbapenemase-producing *E. coli*. J. Antimicrob. Chemother. 75:766. doi: 10.1093/jac/dkz470 31819979

[ref17] Fang Q. Feng Y. Mcnally A. Zong Z. (2022). Characterization of phage resistance and phages capable of intestinal decolonization of carbapenem-resistant *Klebsiella pneumoniae* in mice. Commun Biol 5:48. doi: 10.1038/s42003-022-03001-y, PMID: 35027665 PMC8758719

[ref18] Febvre H. P. Rao S. Gindin M. Goodwin N. D. M. Finer E. Vivanco J. S.. (2019). PHAGE study: Effects of supplemental bacteriophage intake on inflammation and gut microbiota in healthy adults. Nutrients 11:666. doi: 10.3390/nu11030666 30897686 PMC6471193

[ref19] Feng Y. Liu L. Lin J. Ma K. Long H. Wei L.. (2019). Key evolutionary events in the emergence of a globally disseminated, carbapenem resistant clone in the *Escherichia coli* ST410 lineage. Commun. Biol. 2:322. doi: 10.1038/s42003-019-0569-1, PMID: 31482141 PMC6715731

[ref20] Foley S. E. Tuohy C. Dunford M. Grey M. J. De Luca H. Cawley C.. (2021). Gut microbiota regulation of P-glycoprotein in the intestinal epithelium in maintenance of homeostasis. Microbiome 9:183. doi: 10.1186/s40168-021-01137-3, PMID: 34493329 PMC8425172

[ref21] Freires I. A. Sardi J. C. De Castro R. D. Rosalen P. L. (2017). Alternative animal and non-animal models for drug discovery and development: Bonus or burden? Pharm. Res. 34, 681–686. doi: 10.1007/s11095-016-2069-z 27858217

[ref22] Harsanyi E. Juhasz C. Kovacs E. Huzsvai L. Pinter R. Fekete G.. (2020). Evaluation of organic wastes as substrates for rearing *Zophobas morio*, *Tenebrio molitor*, and *Acheta domesticus* larvae as alternative feed supplements. Insects 11:604. doi: 10.3390/insects11090604, PMID: 32899592 PMC7564407

[ref23] Hilty M. Betsch B. Y. Bogli-Stuber K. Heiniger N. Stadler M. Kuffer M.. (2012). Transmission dynamics of extended-spectrum β-lactamase-producing *Enterobacteriaceae* in the tertiary care hospital and the household setting. Clin. Infect. Dis. 55, 967–975. doi: 10.1093/cid/cis581, PMID: 22718774 PMC3436924

[ref24] Hong Y. Thimmapuram J. Zhang J. Collings C. K. Bhide K. Schmidt K.. (2016). The impact of orally administered phages on host immune response and surrounding microbial communities. Bacteriophage 6:e1211066. doi: 10.1080/21597081.2016.1211066, PMID: 27738553 PMC5056770

[ref25] Javaudin F. Bemer P. Batard E. Montassier E. (2021). Impact of phage therapy on multidrug-resistant *Escherichia coli* intestinal carriage in a murine model. Microorganisms 9:2580. doi: 10.3390/microorganisms9122580 34946183 PMC8708983

[ref26] Kim B. R. Shin J. Guevarra R. Lee J. H. Kim D. W. Seol K. H.. (2017). Deciphering diversity indices for a better understanding of microbial communities. J. Microbiol. Biotechnol. 27, 2089–2093. doi: 10.4014/jmb.1709.09027, PMID: 29032640

[ref27] Laird T. Abraham R. Sahibzada S. Abraham S. O’dea M. (2022). *In vitro* demonstration of targeted phage therapy and competitive exclusion as a novel strategy for decolonization of extended-Spectrum-cephalosporin-resistant *Escherichia coli*. Appl. Environ. Microbiol. 88:e0227621. doi: 10.1128/aem.02276-21, PMID: 35254097 PMC9004402

[ref28] Lange A. Schafer A. Frick J. S. (2019). A *Galleria mellonella* oral administration model to study commensal-induced innate immune responses. J. Vis. Exp. doi: 10.3791/59270-v 30958466

[ref29] Lin D. M. Koskella B. Lin H. C. (2017). Phage therapy: an alternative to antibiotics in the age of multi-drug resistance. World J. Gastrointest. Pharmacol. Ther. 8, 162–173. doi: 10.4292/wjgpt.v8.i3.162, PMID: 28828194 PMC5547374

[ref30] Ling W. Furuya-Kanamori L. Ezure Y. Harris P. N. A. Paterson D. L. (2021). Adverse clinical outcomes associated with infections by Enterobacterales producing ESBL (ESBL-E): a systematic review and meta-analysis. JAC Antimicrob. Resist. 3:dlab068. doi: 10.1093/jacamr/dlab068, PMID: 35233528 PMC8210200

[ref31] Linkevicius M. Bonnin R. A. Alm E. Svartstrom O. Apfalter P. Hartl R.. (2023). Rapid cross-border emergence of NDM-5-producing *Escherichia coli* in the European Union/European economic area, 2012 to June 2022. Euro Surveill. 28:2300209. doi: 10.2807/1560-7917.ES.2023.28.19.2300209, PMID: 37166762 PMC10176832

[ref32] Luo L. Wang Y. Guo H. Yang Y. Qi N. Zhao X.. (2021). Biodegradation of foam plastics by *Zophobas atratus* larvae (Coleoptera: Tenebrionidae) associated with changes of gut digestive enzymes activities and microbiome. Chemosphere 282:131006. doi: 10.1016/j.chemosphere.2021.131006, PMID: 34118623

[ref33] Mason C. J. St Clair A. Peiffer M. Gomez E. Jones A. G. Felton G. W.. (2020). Diet influences proliferation and stability of gut bacterial populations in herbivorous lepidopteran larvae. PLoS One 15:e0229848. doi: 10.1371/journal.pone.0229848, PMID: 32168341 PMC7069608

[ref34] Mcmurdie P. J. Holmes S. (2013). Phyloseq: an R package for reproducible interactive analysis and graphics of microbiome census data. PLoS One 8:e61217. doi: 10.1371/journal.pone.0061217, PMID: 23630581 PMC3632530

[ref35] Mirza K. A. Nietzsche S. Makarewicz O. Pletz M. W. Thieme L. (2024). Bacteriophage-mediated decolonization of *Klebsiella pneumoniae* in a novel *Galleria mellonella* gut colonization model with *Enterobacteriaceae*. Sci. Rep. 14:318. doi: 10.1038/s41598-023-50823-9, PMID: 38172281 PMC10764950

[ref36] Moor J. Wuthrich T. Aebi S. Mostacci N. Overesch G. Oppliger A.. (2021). Influence of pig farming on human gut microbiota: role of airborne microbial communities. Gut Microbes 13, 1–13. doi: 10.1080/19490976.2021.1927634, PMID: 34060426 PMC8172160

[ref37] Moser A. I. Campos-Madueno E. I. Perreten V. Endimiani A. (2022). Genome stability during serial subculturing in hyperepidemic multidrug-resistant *Klebsiella pneumoniae* and *Escherichia coli*. J. Glob. Antimicrob. Resist. 31, 152–161. doi: 10.1016/j.jgar.2022.08.014, PMID: 36049731

[ref38] Moser A. I. Campos-Madueno E. I. Sendi P. Perreten V. Keller P. M. Ramette A.. (2021). Repatriation of a patient with COVID-19 contributed to the importation of an emerging carbapenemase producer. J. Glob. Antimicrob. Resist. 27, 267–272. doi: 10.1016/j.jgar.2021.10.012, PMID: 34718203 PMC8552635

[ref39] Nigg A. Brilhante M. Dazio V. Clement M. Collaud A. Gobeli Brawand S.. (2019). Shedding of OXA-181 carbapenemase-producing *E. coli* from companion animals after hospitalisation in Switzerland: an outbreak in 2018. Euro Surveill.:24. doi: 10.2807/1560-7917.ES.2019.24.39.1900071 PMC6774230 31576806

[ref40] Olsson L. M. Boulund F. Nilsson S. Khan M. T. Gummesson A. Fagerberg L.. (2022). Dynamics of the normal gut microbiota: a longitudinal one-year population study in Sweden. Cell Host Microbe 30:e723, –739.e3. doi: 10.1016/j.chom.2022.03.002 35349787

[ref41] Peirano G. Pitout J. D. D. (2019). Extended-Spectrum β-lactamase-producing Enterobacteriaceae: update on molecular epidemiology and treatment options. Drugs 79, 1529–1541. doi: 10.1007/s40265-019-01180-3, PMID: 31407238

[ref42] Perez F. Pultz M. J. Endimiani A. Bonomo R. A. Donskey C. J. (2011). Effect of antibiotic treatment on establishment and elimination of intestinal colonization by KPC-producing *Klebsiella pneumoniae* in mice. Antimicrob. Agents Chemother. 55, 2585–2589. doi: 10.1128/AAC.00891-10, PMID: 21422202 PMC3101444

[ref43] Pitout J. D. D. Finn T. J. (2020). The evolutionary puzzle of *Escherichia coli* ST131. Infect. Genet. Evol. 81:104265. doi: 10.1016/j.meegid.2020.104265, PMID: 32112974

[ref44] Pitout J. D. D. Peirano G. Matsumura Y. Devinney R. Chen L. (2024). *Escherichia coli* sequence type 410 with carbapenemases: a paradigm shift within *E. coli* toward multidrug resistance. Antimicrob. Agents Chemother. 68:e0133923. doi: 10.1128/aac.01339-23 38193668 PMC10869336

[ref45] Rinninella E. Raoul P. Cintoni M. Franceschi F. Miggiano G. A. D. Gasbarrini A.. (2019). What is the healthy gut microbiota composition? A changing ecosystem across age, environment, diet, and diseases. Microorganisms 7:14. doi: 10.3390/microorganisms7010014, PMID: 30634578 PMC6351938

[ref46] Roer L. Overballe-Petersen S. Hansen F. Schonning K. Wang M. Roder B. L.. (2018). *Escherichia coli* sequence type 410 is causing new international high-risk clones. mSphere. 3, e00337–18. doi: 10.1128/mSphere.00337-18 30021879 PMC6052333

[ref47] Rumbos C. I. Athanassiou C. G. (2021). The Superworm, *Zophobas morio* (Coleoptera:Tenebrionidae): a 'Sleeping Giant' in nutrient sources. J. Insect Sci. 21:13. doi: 10.1093/jisesa/ieab014, PMID: 33834209 PMC8033247

[ref48] Schmidt J. S. Kuster S. P. Nigg A. Dazio V. Brilhante M. Rohrbach H.. (2020). Poor infection prevention and control standards are associated with environmental contamination with carbapenemase-producing Enterobacterales and other multidrug-resistant bacteria in Swiss companion animal clinics. Antimicrob. Resist. Infect. Control 9:93. doi: 10.1186/s13756-020-00742-5, PMID: 32576281 PMC7310346

[ref49] Seiffert S. N. Hilty M. Kronenberg A. Droz S. Perreten V. Endimiani A. (2013). Extended-spectrum cephalosporin-resistant *Escherichia coli* in community, specialized outpatient clinic and hospital settings in Switzerland. J. Antimicrob. Chemother. 68, 2249–2254. doi: 10.1093/jac/dkt208, PMID: 23759671

[ref50] Silva A. Silva V. Pereira J. E. Maltez L. Igrejas G. Valentao P.. (2023). Antimicrobial resistance and clonal lineages of *Escherichia coli* from food-producing animals. Antibiotics (Basel) 12:1061. doi: 10.3390/antibiotics12061061 37370379 PMC10295564

[ref51] Singkum P. Suwanmanee S. Pumeesat P. Luplertlop N. (2019). A powerful *in vivo* alternative model in scientific research: *Galleria mellonella*. Acta Microbiol. Immunol. Hung. 66, 31–55. doi: 10.1556/030.66.2019.001, PMID: 30816806

[ref52] Sneddon L. U. Halsey L. G. Bury N. R. (2017). Considering aspects of the 3Rs principles within experimental animal biology. J. Exp. Biol. 220, 3007–3016. doi: 10.1242/jeb.147058, PMID: 28855318

[ref53] Stercz B. Farkas F. B. Toth A. Gajdacs M. Domokos J. Horvath V.. (2021). The influence of antibiotics on transitory resistome during gut colonization with CTX-M-15 and OXA-162 producing *K. pneumoniae* ST15. Sci. Rep. 11:6335. doi: 10.1038/s41598-021-85766-6, PMID: 33737655 PMC7973416

[ref54] Von Bredow Y. M. Prochazkova P. Dvorak J. Skanta F. Trenczek T. E. Bilej M.. (2023). Differential expression of immunity-related genes in larval *Manduca sexta* tissues in response to gut and systemic infection. Front. Cell. Infect. Microbiol. 13:1258142. doi: 10.3389/fcimb.2023.1258142, PMID: 37900309 PMC10603244

[ref55] Walters W. Hyde E. R. Berg-Lyons D. Ackermann G. Humphrey G. Parada A.. (2016). Improved bacterial 16S rRNA gene (V4 and V4-5) and fungal internal transcribed spacer marker gene primers for microbial community surveys. mSystems. 1, e00009–e00015. doi: 10.1128/mSystems.00009-15 PMC5069754 27822518

[ref56] Wang S. Mu L. Yu C. He Y. Hu X. Jiao Y.. (2024). Microbial collaborations and conflicts: unraveling interactions in the gut ecosystem. Gut Microbes 16:2296603. doi: 10.1080/19490976.2023.2296603, PMID: 38149632 PMC10761165

[ref57] Zhang X. Zhao Y. Wu Q. Lin J. Fang R. Bi W.. (2019). Zebrafish and *Galleria mellonella*: models to identify the subsequent infection and evaluate the immunological differences in different *Klebsiella pneumoniae* intestinal colonization strains. Front. Microbiol. 10:2750. doi: 10.3389/fmicb.2019.02750, PMID: 31849893 PMC6900958

[ref58] Zschach H. Joensen K. G. Lindhard B. Lund O. Goderdzishvili M. Chkonia I.. (2015). What can we learn from a metagenomic analysis of a georgian bacteriophage cocktail? Viruses 7, 6570–6589. doi: 10.3390/v7122958, PMID: 26703713 PMC4690881

