# Supplementary material for: A new in vivo model of intestinal colonization using Zophobas morio larvae: testing hyperepidemic ESBL- and carbapenemase-producing Escherichia coli clones
Source: Front Microbiol. 2024 Apr 10;15:1381051. doi: 10.3389/fmicb.2024.1381051 (PMC11039899; doi:10.3389/fmicb.2024.1381051)
Supplement: Supplementary file 1 [file Data_Sheet_1.PDF]

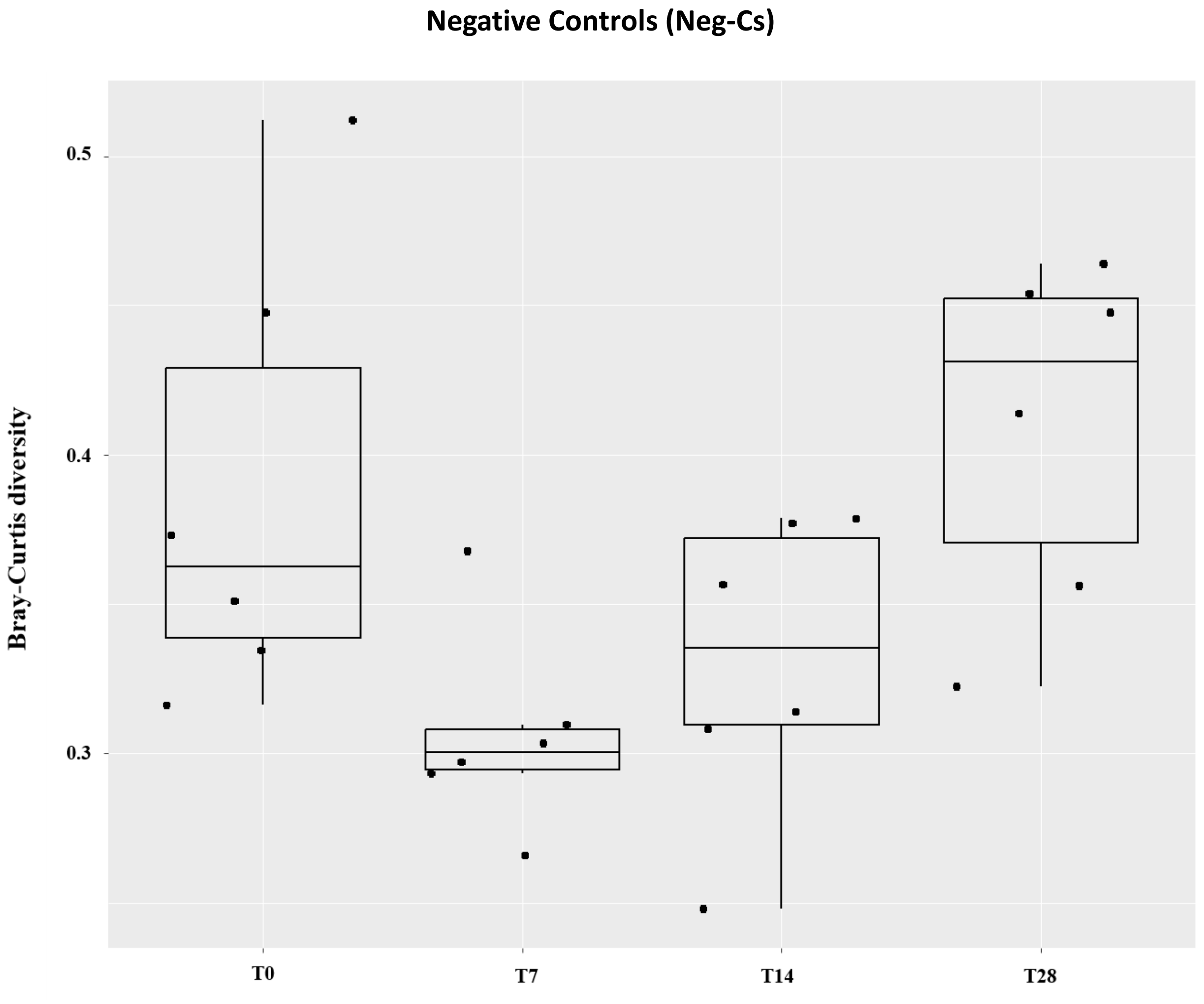

Figure S1. Beta diversity analysis based on the Bray-Curtis dissimilarity index (BCDI) of the 4 negative controls (Neg-Cs) indicated in Figure 4. Values were obtained comparing the microbial community composition between the samples (i.e., #1 *vs.* #2, #1 *vs.* #3, #1 *vs.* #4, #2 *vs.* #3, #2 *vs.* #4, #3 *vs.* #4) based on the entire ASV data obtained. The box plot represent the mean of the four experiments (#1, #2, #3 and #4) at every timepoint (T0, T7, T14 and T28). Box-and-whisker plots represent the median and interquartile range of the BCDI distance between samples.

Ec-4901.28

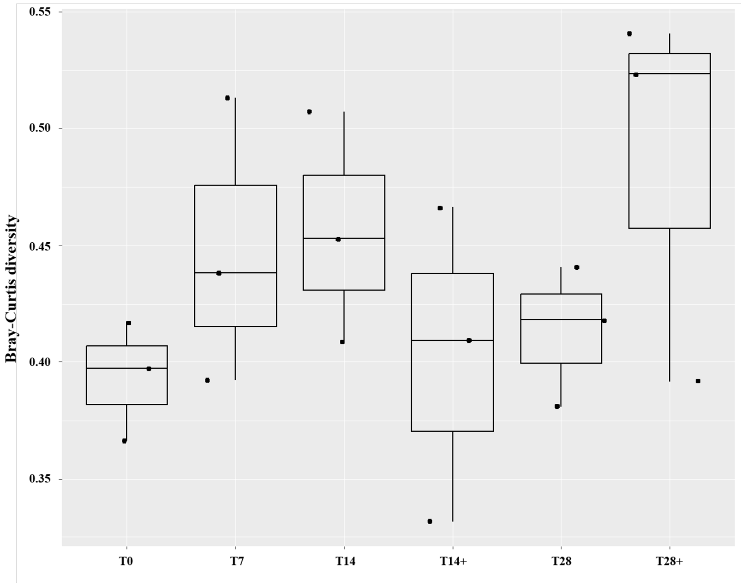

Ec-042

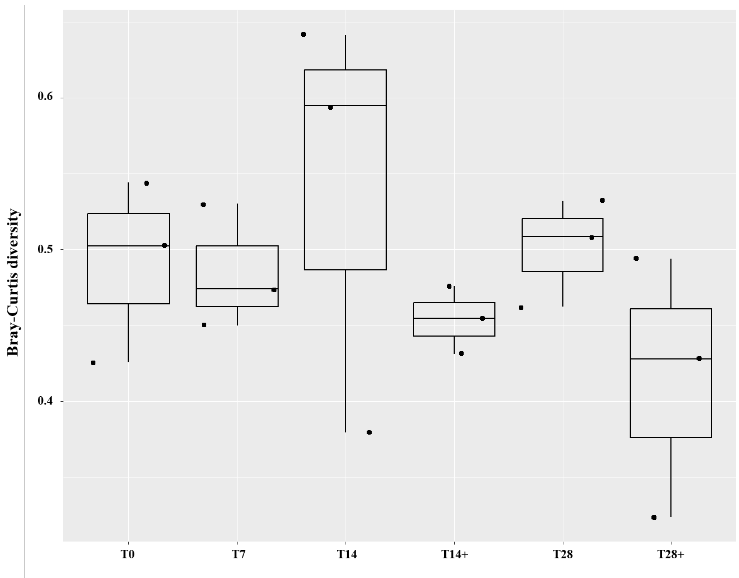

Ec-050

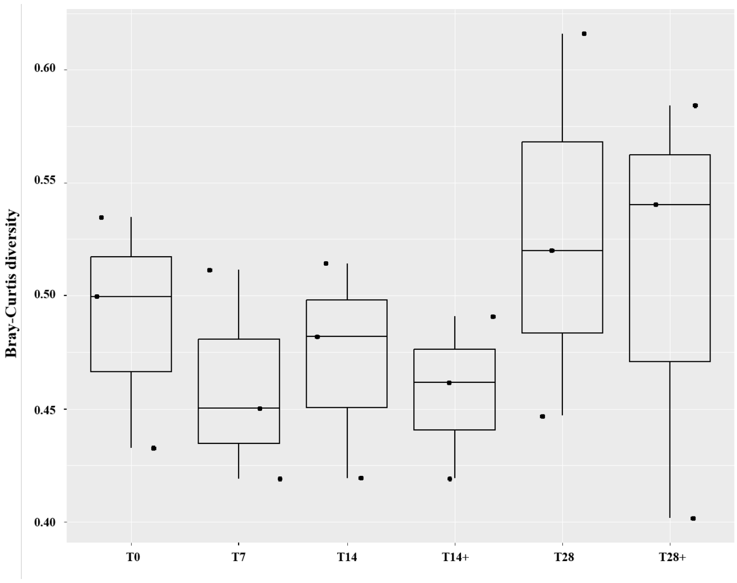

Figure S2. Beta diversity analysis based on the Bray-Curtis dissimilarity index (BCDI) of the 3 experiments (A, B, and C indicated in Figure 5). Values were obtained comparing the microbial community composition between the samples (i.e., A *vs.* B, A *vs.* C, and B *vs.* C) based on the entire ASV data obtained. The box plot represent the mean of the three experiments (A, B, and C) at every timepoint (T0, T7, T14, T14+, T28 and T28+). Box-and-whisker plots represent the median and interquartile range of the BCDI distance between samples.

Table S1. Data from all *in vivo* experiments expressed in colony-forming units (CFU)/mL or plaques-forming units (PFU)/mL.

MDR-*Ec* strain	Groups	Experiment # / 16S rRNA analysis group	CFU/mL or PFU/mL							
			T0	T2	T4	T7	T10	T14- or T14+	T21- or T21+	T28- or T28+
Ec-4901.28	Non-treated	#1	0.00E+00	1.13E+06	1.63E+07	1.30E+07	1.37E+04	1.61E+04	NP	NP
		#2	0.00E+00	1.19E+05	7.88E+05	1.76E+06	2.30E+05	1.40E+05	NP	NP
		#3	0.00E+00	4.49E+06	NP	1.63E+06	NP	4.14E+03	5.00E+02	2.35E+03
		#4	0.00E+00	9.26E+06	NP	3.70E+06	NP	NP	NP	NP
		#5	0.00E+00	6.03E+06	1.00E+07	8.10E+06	NP	NP	NP	NP
		#6 / A [a]	0.00E+00	4.55E+06	1.67E+07	5.96E+06	1.79E+04	1.03E+05	1.64E+04	4.71E+03
		#7 / B	0.00E+00	2.87E+05	2.94E+07	2.86E+06	7.62E+02	8.47E+05	1.89E+05	1.26E+05
		#8 / C	0.00E+00	1.48E+06	2.51E+07	9.05E+06	6.27E+04	2.24E+02	2.73E+03	0.00E+00
		#9	0.00E+00	2.51E+06	6.16E+06	1.65E+07	8.96E+04	6.40E+04	4.93E+02	1.20E+05
		**Mean**	**0.00E+00**	**3.32E+06**	**1.49E+07**	**6.95E+06**	**6.91E+04**	**1.68E+05**	**4.18E+04**	**5.07E+04**
	Receiving bacteriophages	#10 (= #6 until T7) / A					2.16E+05	8.01E+04	0.00E+00	0.00E+00
		#11 (= #7 until T7) / B					1.08E+03	4.57E+03	5.69E+03	0.00E+00
		#12 (= #9 until T7) / C					4.93E+04	1.41E+03	6.12E+05	NP
		**Mean**					**8.88E+04**	**2.87E+04**	**2.06E+05**	**0.00E+00**
	Receiving 1X dPBS (control)	#13 (= #8 until T7)					1.03E+05	1.63E+05	6.14E+05	2.03E+05
		#14 (= #9 until T7)					1.34E+04	8.61E+03	5.37E+04	2.24E+03
		**Mean**					**5.83E+04**	**8.57E+04**	**3.34E+05**	**1.03E+05**
	Viral titer	#10				0.00E+00	4.20E+02	1.05E+03	2.10E+02	0.00E+00
		#11				0.00E+00	9.20E+02	3.54E+04	1.10E+02	0.00E+00
		#12				0.00E+00	2.10E+02	0.00E+00	0.00E+00	NP
		**Mean**				**0.00E+00**	**5.17E+02**	**1.22E+04**	**1.07E+02**	**0.00E+00**
Ec-042	Non-treated	#1 / A	0.00E+00	5.50E+06	3.99E+06	7.06E+06	2.82E+05	1.09E+05	0.00E+00	8.78E+03
		#2 / B	0.00E+00	3.16E+07	7.93E+06	2.38E+07	4.48E+03	5.89E+06	0.00E+00	2.16E+04
		#3 / C	0.00E+00	7.90E+06	2.17E+07	1.21E+06	1.26E+05	0.00E+00	0.00E+00	0.00E+00
		#4	0.00E+00	4.33E+06	2.24E+07	4.80E+06	9.86E+04	2.11E+05	4.76E+04	0.00E+00
		**Mean**	**0.00E+00**	**1.23E+07**	**1.40E+07**	**9.22E+06**	**1.28E+05**	**1.55E+06**	**1.19E+04**	**7.60E+03**
	Receiving bacteriophages	#5 (=#1 until T7) / A					8.52E+04	1.21E+03	0.00E+00	1.84E+03
		#6 (=#2 until T7) / B					1.79E+04	2.97E+04	NP	1.88E+05
		#7 (=#3 until T7) / C					1.50E+05	3.14E+03	0.00E+00	0.00E+00
		**Mean**					**8.44E+04**	**1.14E+04**	**0.00E+00**	**6.33E+04**
	Receiving 1X dPBS (control)	#8 (= #3 until T7)					2.15E+05	2.11E+03	1.34E+02	1.87E+05
		#9 (= #4 until T7)					7.53E+05	1.62E+05	0.00E+00	0.00E+00
		**Mean**					**4.84E+05**	**8.19E+04**	**6.70E+01**	**9.35E+04**
	Viral titer	#5				0.00E+00	0.00E+00	0.00E+00	0.00E+00	0.00E+00
		#6				0.00E+00	2.10E+02	6.30E+02	0.00E+00	0.00E+00
		#7				0.00E+00	0.00E+00	0.00E+00	NP	NP
		**Mean**				**0.00E+00**	**7.00E+01**	**2.10E+02**	**0.00E+00**	**0.00E+00**
Ec-050	Non-treated	#1 / A	0.00E+00	4.42E+06	1.56E+07	3.59E+06	1.12E+05	3.14E+02	4.48E+01	0.00E+00
		#2 / B	0.00E+00	1.82E+06	4.20E+06	6.55E+06	0.00E+00	1.90E+04	5.60E+04	0.00E+00
		#3 / C	0.00E+00	2.08E+06	1.73E+06	2.86E+07	3.14E+03	3.33E+03	0.00E+00	0.00E+00
		#4	0.00E+00	2.08E+06	1.73E+06	2.86E+07	NP	NP	NP	NP
		#5	0.00E+00	3.23E+06	1.11E+07	1.84E+07	2.24E+04	3.00E+03	1.03E+03	0.00E+00
		**Mean**	**0.00E+00**	**2.73E+06**	**6.87E+06**	**1.71E+07**	**3.44E+04**	**6.41E+03**	**1.43E+04**	**0.00E+00**
	Receiving bacteriophages	#6 (= #1 until T7) / A					1.79E+04	6.27E+02	2.69E+02	7.85E+04
		#7 (= #2 until T7) / B					4.48E+03	6.11E+04	8.56E+03	7.13E+02
		#8 (= #3 until T7) / C					1.03E+03	2.24E+02	9.52E+04	0.00E+00
		**Mean**					**7.80E+03**	**2.07E+04**	**3.47E+04**	**2.64E+04**
	Receiving 1X dPBS (control)	#9 (= #4 until T7)					2.38E+03	0.00E+00	0.00E+00	0.00E+00
		#10 (= #5 until T7)					0.00E+00	4.48E+01	0.00E+00	0.00E+00
		**Mean**					**1.19E+03**	**2.24E+01**	**0.00E+00**	**0.00E+00**
	Viral titer	#6				0.00E+00	0.00E+00	6.60E+02	0.00E+00	0.00E+00
		#7				0.00E+00	1.10E+02	1.10E+03	3.30E+02	0.00E+00
		#8				0.00E+00	0.00E+00	0.00E+00	NP	NP
		**Mean**				**0.00E+00**	**3.67E+01**	**5.87E+02**	**1.65E+02**	**0.00E+00**

Note. NP, not performed; T14- / T21- / T28-, larvae not receiving bacteriophages; T14+ / T21+/ T28+, larvae receiving the *INTESTI bacteriophage* cocktail.

[a] For this experiment (only), larvae were still colonized with 1.19×10^4 CFU/mL after 35 days.

Table S2. Time growth assay for the 3 multidrug-resistant *E. coli* (MDR-*Ec*) strains in Luria-Bertani (LB) broth without and with 20% 1xPBS

**MDR-*Ec***		**Growth in LB broth (CFU/mL)**					**Growth in LB broth with 20% 1xPBS (CFU/mL)**				
		**2 hrs**	**4 hrs**	**6 hrs**	**8 hrs**	**24 hrs**	**2 hrs**	**4 hrs**	**6 hrs**	**8 hrs**	**24 hrs**
***Ec*-4901.28**	Mean	$1.9x10^5$	$4.8x10^6$	$3.0x10^8$	$5.2x10^8$	$4.7x10^8$	$4.7x10^5$	$4.6x10^6$	$1.2x10^8$	$3.7x10^8$	$6.3x10^8$
	Run 1	$1.1x10^5$	$1.2x10^7$	$7.5x10^8$	NP	NP	$4.0x10^5$	$1.0x10^7$	$2.0x10^8$	NP	NP
	Run 2	$3.9x10^5$	$1.9x10^6$	$1.3x10^8$	$5.2x10^8$	$5.6x10^8$	$1.0x10^6$	$2.9x10^6$	$1.7x10^8$	$4.4x10^8$	$1.1x10^9$
	Run 3	$8.0x10^3$	$1.0x10^5$	$2.6x10^7$	NP	NP	$1.3x10^4$	$5.0x10^5$	$2.8x10^6$	$3.0x10^8$	$4.3x10^8$
	Run 4	NP	NP	NP	NP	$3.8x10^8$	NP	NP	NP	NP	$3.6x10^8$
***Ec*-042**	Mean	$5.4x10^4$	$2.0x10^6$	$3.0x10^7$	$7.5x10^7$	$6.3x10^8$	$6.6x10^4$	$1.5x10^6$	$1.4x10^8$	$2.5x10^8$	$4.2x10^8$
	Run 1	$5.0x10^4$	$5.0x10^6$	$9.0x10^7$	NP	NP	$1.3x10^5$	$4.1x10^6$	$2.2x10^8$	NP	NP
	Run 2	$2.0x10^3$	$3.0x10^5$	$6.0x10^5$	$5.0x10^7$	$4.9x10^8$	$2.0x10^3$	$1.0x10^5$	$1.2x10^6$	$1.0x10^8$	$4.0x10^8$
	Run 3	$1.1x10^5$	$7.0x10^5$	$1.9x10^6$	$1.0x10^8$	$1.0x10^9$	$6.7x10^4$	$5.0x10^5$	$2.2x10^8$	$4.0x10^8$	$4.0x10^8$
	Run 4	NP	NP	NP	NP	$4.1x10^8$	NP	NP	NP	NP	$4.7x10^8$
***Ec*-050**	Mean	$1.6x10^5$	$5.4x10^6$	$1.3x10^8$	$4.1x10^8$	$7.1x10^8$	$1.7x10^5$	$5.1x10^6$	$1.2x10^8$	$3.0x10^8$	$3.2x10^8$
	Run 1	$2.0.x10^5$	$6.1x10^6$	$1.2x10^8$	$1.5x10^8$	NP	$1.4x10^5$	$7.9x10^6$	$9.0x10^7$	$1.2x10^8$	NP
	Run 2	$2.4x10^5$	$5.6x10^6$	$1.8x10^8$	$4.8x10^8$	$1.3x10^9$	$2.4x10^5$	$4.4x10^6$	$1.8x10^8$	$3.7x10^8$	$4.0x10^8$
	Run 3	$6.1x10^5$	$4.7x10^6$	$1.0x10^8$	$6.0x10^8$	NP	$1.3x10^5$	$3.1x10^6$	$1.1x10^8$	$4.0x10^8$	NP
	Run 4	NP	NP	NP	NP	$3.7x10^8$	NP	NP	NP	NP	$2.6x10^8$
	Run 5	NP	NP	NP	NP	$4.0x10^8$	NP	NP	NP	NP	$3.2x10^8$

Note. CFU, colony forming unit; NP, not performed

MATERIALS AND METHODS (IN BRIEF)

The 3 MDR-*Ec* strains were grown overnight in MacConkey II plate (Becton-Dickinson). Fresh colonies were diluted in sterile 0.85% NaCl to obtain an inoculum of 0.5 McFarland (1.5 x 10^8 CFU/mL). Then, 5 µL were diluted at the ratio of 1:1000 in 5 mL Luria-Bertani (LB) broth +20% 1xPBS (Thermo Fisher Scientific). A growth control of the 3 strains was also performed by adding the 5 µL to 5 mL LB broth. Tubes were incubated at 36±1°C.

The bacterial growth was monitored at time intervals of 0, 2, 4, 6, 8, and 24 hrs, using appropriate serial dilutions of aliquots in 0.85% NaCl. Then, 100 µL of the suspension was plated in MacConkey agar plates and incubated for 24 hrs at 36±1°C. Colony count (CFU/mL) was determined for every MDR-*Ec* strain. Data were analyzed using two-way ANOVA followed by Tukey post hoc from Graph Pad Prism version 9 for Windows (Graph Pad Software Inc., San Diego, CA, USA).

Table S3. Results of SNV analyses comparing chromosome/plasmids of the starting (T0) multidrug-resistant *E. coli* (MDR-*Ec*) to the strains recovered at the end of colonization experiments.

SNVs analysis		Chromosomal SNVs		Plasmid SNVs		
Original MDR-*Ec*	MDR-*Ec* at the end of experiment # [a]	Indels No.	Target *CDS*	Plasmid	Indels No.	Target *CDS*
***Ec*-4901.28**	***Ec*-49_e9_T28**	6	IS*3*-like	P1 (for both strains)	10	ArgR family transcriptional regulator
		3	Non-coding region		2	Non-coding region
		2	IS*3*		1	IS*5*
		1	*sapB*		1	DUF1281 domain-containing protein
		1	IS*1*		1	DsbA family oxidoreductase
		1	*IucA*/*IucC* family siderophore biosynthesis		1	*sopA*
	***Ec*-49_e11_T21+**	15	EntS/YbdA MFS transporter		1	*sbb*
		6	IS*3*-like		1	hypothetical protein
		4	Non-coding region		1	*crcB*
		2	IS*1*		1	*RepB*
		1	*IucA*/*IucC* family siderophore biosynthesis			
		1	Phage tail protein			
***Ec*-042**	***Ec*-042_e2_T28**	1	IS*As1*	P1	None	
		1	IS*3*-like	P2	Insertion of 834bp [b]	*pilV*
		1	IS*30*-like		Insertion of 1948bp [c]	hypothetical protein (position 32584-34532)
		1	Non-coding region			
		1	*tuf*			
	***Ec*-042_e6_T28+**	14	RHS repeat protein	P1	None	
		3	Non-coding region	P2	Insertion of 1416bp [d]	hypothetical protein (position 31749-33165)
		2	IS*1*-like			
		2	16S rRNA			
		1	IS*As1*			
		1	IS*3*-like			
		1	IS*30*-like			
		1	*tuf*			
		1	23S rRNA			
***Ec*-050**	***Ec*-050_e2_T21**	2	Glutamate decarboxylase	P1	None	
		3	IS*66*-like	P2	1	IS*6*-like
		3	glnS glutamine tRNA ligase	P3	None	
		1	Peptidase S74			
		1	IS*4*-like			
		1	AsmA2 domain-containing protein			
		1	*tuf*			
		1	Non-coding region			
	***Ec*-050_e7_T28+**	3	IS*66*-like	P1	1	IS*6*-like
		3	glnS glutamine tRNA ligase	P2	1	IS*6*-like
		3	IS*66*-like	P3	None	
		2	Glutamate decarboxylase			
		1	IS*1*-like			
		1	IS*4*-like			
		1	AsmA2 domain-containing protein			
		1	*tuf*			
		1	Non-coding region			

[a] T21+ and T28+, strains recovered during experiments with administration of bacteriophages

[b] Most similar gene was hypothetical protein (identity 100%): GenBank: WP_232203411.1

[c] Most similar gene was shufflon protein D' (identity 100%): GenBank: WHF95111.1

[d] Most similar gene was shufflon protein D' (identity 100%): GenBank: WHF95111.1
